# Rapid plant functional trait responses to warming, flooding, and herbivory in high-latitude coastal wetlands

**DOI:** 10.1007/s00442-026-05876-8

**Published:** 2026-03-08

**Authors:** Cristina Chirvasa, Matteo Petit Bon, Kelvyn K. Bladen, Katharine C. Kelsey, A. Joshua Leffler, Tyler J. Williams, Karen H. Beard

**Affiliations:** 1 Department of Wildland Resources and Ecology Center, Utah State University, Logan, UT USA; 2 Department of Applied Ecology, North Carolina State University, Raleigh, NC USA; 3 Department of Mathematics and Statistics, Utah State University, Logan, UT USA; 4 Department of Geography & Environmental Science, University of Colorado Denver, Denver, CO USA; 5 Department of Natural Resource Management, South Dakota State University, Brookings, SD USA

**Keywords:** Arctic, Leaf economics spectrum, Phenotypic plasticity, Plant size, Yukon-Kuskokwim Delta (Alaska)

## Abstract

Climate change is rapidly altering high-latitude coastal wetlands through increasing temperatures, more frequent flooding, and changing herbivore abundance and distribution. Examining plant functional trait responses to these drivers provides insight into how plant communities are shaped by climate change. We used a one-year full-factorial mesocosm experiment in coastal Alaska to assess immediate responses of two size-related traits (vegetative height and leaf area) and two leaf economics traits (specific leaf area [SLA] and leaf dry matter content [LDMC]) to warming, flooding, and goose herbivory in three dominant species across two wetland communities (Lowland and Upland) differing in landscape position. We sampled different sedges (*Carex rariflora* and *Carex lyngbyei*) and the same deciduous dwarf-shrub (*Salix fuscescens*) in the two wetlands. Warming increased plant size for the sedge (leaf area: + 15%) and the shrub (height: + 15%; leaf area: + 19%) in the Lowland wetland only. Flooding promoted acquisitive trait values for the Lowland sedge (SLA: + 8%; LDMC: − 6%) and conservative values for the Upland shrub (SLA: − 9%; LDMC: + 11%). The shrub thus showed clear wetland-specific responses to warming and flooding. Herbivory triggered coordinated responses across all three species, consistently decreasing size-related trait values (height: − 17–35%; leaf area: − 33–50%) and promoting acquisitive trait values (SLA: + 14–40%; LDMC: − 12–23%). Despite the absence of interactions, each driver contributed significantly to trait variation, underscoring the value of multifactorial approaches. The combination of consistent (herbivory) and context-dependent (flooding and warming) responses highlights the complexity of trait responses and improves predictions of rapid phenotypic adjustments in coastal high-latitude wetlands.

**Supplementary Information:**

The online version contains supplementary material available at 10.1007/s00442-026-05876-8.

## Introduction

Coastal high-latitude regions are undergoing rapid climate change (Irrgang et al. 2022; IPCC 2023). The Arctic as a whole has warmed nearly four times faster than the global average (Rantanen et al. 2022). Concurrently, coastal flooding has intensified (Kirezci et al. 2020) due to accelerated sea-level rise (Hamlington et al. 2024), diminishing sea ice extent and increased storm activity (Vermaire et al. 2013), land subsidence (Nicholls et al. 2021), and more frequent, intense cyclones (Parker et al. 2022). At the same time, goose herbivore pressure is shifting as warming advances migration phenology, overall abundances are increasing, and rising coastal flooding is likely driving geese toward previously less utilized habitats further inland (Fox and Madsen 2017; Lameris et al. 2021; Koltz et al. 2022). Understanding how these concurrent global change drivers influence plant communities in high-latitude coastal ecosystems is critical for anticipating ecological change in these rapidly transforming regions.

A deeper understanding of climate change impacts on plant communities can be gained by examining how functional traits respond to environmental change (Suding et al. 2008; Myers‐Smith et al. 2019), as these traits mediate species’ interactions with their environment and determine their capacity to adjust to new conditions (Violle et al. 2007). Plants with greater trait plasticity are generally better able to cope with changing environments, while those with limited capacity for phenotypic adjustment may be more vulnerable (Nicotra et al. 2010; Henn et al. 2018). Aboveground functional trait variation is primarily structured along two axes: plant size and the leaf economics spectrum (Díaz et al. 2016). Size-related traits such as vegetative height and leaf area are key to light competition and reproductive success (Pérez-Harguindeguy et al. 2013; Beccari and Carmona 2024). In contrast, the leaf economics axis reflects a trade-off between rapid resource acquisition and efficient resource conservation (Wright et al. 2004; Reich 2014). It is commonly captured by specific leaf area (SLA) and leaf dry matter content (LDMC), with high SLA and low LDMC indicating acquisitive strategies (fast growth, high photosynthetic rates) and the opposite combination indicating conservative strategies (resource retention, stress tolerance). Investigating how these trait dimensions respond to warming, flooding, and herbivory in high-latitude coastal plant communities may help predict functional and ecosystem-level consequences of ongoing environmental change.

Low temperatures make Arctic ecosystems generally temperature-limited, such that even modest warming can alleviate cold constraints and elicit substantial changes in plant growth and associated traits. Warming effects on plant traits in the Arctic are generally well-documented (Myers‐Smith et al. 2019), with consistent evidence for increased height and leaf area (Hudson et al. 2011; Baruah et al. 2017; Bjorkman et al. 2018). Reduced temperature limitation also tends to favor a shift in economics traits toward resource acquisition (Bjorkman et al. 2018; Jessen et al. 2020; Wei et al. 2023), although more conservative responses have also been observed (Hudson et al. 2011). In fact, responses in both size-related and especially economics traits vary with environmental conditions and species identity (Baruah et al. 2017; Betway et al. 2021; Kemppinen and Niittynen 2022). Across the tundra biome, shrubs generally exhibit stronger growth responses to warming than graminoids, particularly in wetter and warmer sites (Elmendorf et al. 2012), and the positive relationship between SLA and temperature is also stronger in wetter environments (Bjorkman et al. 2018). These findings underscore the importance of considering environmental context and co-occurring global change drivers, such as flooding, which might modify the conditions under which warming influences functional trait responses in coastal high-latitude wetlands.

Increased flooding can affect plant functional traits both indirectly, by altering environmental conditions, and directly through physical stress. Depending on whether the benefits (e.g., higher moisture and nutrient availability) outweigh the stresses (e.g., oxygen deprivation, sedimentation, salinity, and mechanical disturbance), flooding may induce contrasting responses in size-related and leaf economic traits. Accordingly, trait responses to flooding have varied across ecosystems, with plant height shown to decrease (Baastrup-Spohr et al. 2015; Fu et al. 2015) or increase (Purcell et al. 2019), and SLA and LDMC either increasing (Jung et al. 2010; Violle et al. 2011) or decreasing (Fu et al. 2015; Purcell et al. 2019). Such divergent outcomes may reflect species-specific differences in flood tolerance. For instance, in high-latitude coastal ecosystems, flooding has been shown to reduce shrub biomass while enhancing graminoid growth (Person and Ruess 2003; Churchill et al. 2015; Petit Bon et al. 2024), suggesting that flood-tolerant, fast-growing graminoids may adopt more acquisitive trait expressions under increased flooding, whereas slower-growing, less flood-tolerant shrubs may display reduced growth and more conservative trait values. As flooding effects on plant traits remain poorly studied in high-latitude coastal ecosystems, further investigation is essential for understanding functional responses to this intensifying global change driver.

In addition to warming and flooding, herbivores can shape plant functional traits by removing biomass through grazing, trampling vegetation and soil, and returning nutrients via feces and urine. Herbivory generally reduces size-related trait values through direct biomass loss, disturbance from trampling, and the selection of larger plants (Díaz et al. 2007; Egelkraut et al. 2020; Barbero-Palacios et al. 2024). Moreover, avoidance-based species—those minimizing the likelihood or intensity of damage—typically display more conservative traits (low SLA and high LDMC). In contrast, grazing-tolerant plants—those capable of compensating or regrowing after tissue loss—tend to express acquisitive traits that support rapid regrowth, often enhanced by nutrient inputs from animal excreta (Díaz et al. 2007; Niu et al. 2016; Jessen et al. 2020). Graminoids, being more grazing-tolerant and capable of rapid regrowth, might thus be expected to shift toward more acquisitive traits, whereas shrubs, which are less able to replace lost tissue, may respond with more conservative trait shifts that reduce further damage. Yet, while geese promote regrowth in preferred forage species such as graminoids (Cargill and Jefferies 1984; Beard et al. 2019), how they affect their functional traits, and how these responses compare to those of the less preferred shrubs, remain unclear. Given the central ecological role of herbivores in high-latitude ecosystems, assessing their influence on plant traits, especially alongside other global change drivers, is a critical research priority (Barrio et al. 2025).

The Yukon-Kuskokwim (Y-K) Delta in western Alaska, one of the largest high-latitude riverine deltas in North America (~ 130,000 km^2^), is undergoing rapid change. The region is warming rapidly (SNAP 2020) and increasingly exposed to frequent flooding, including prolonged overbank flow from monthly high tides and intensified storm events (Jorgenson & Ely 2001; Terenzi et al. 2014). It also serves as a major summer breeding ground for hundreds of thousands of migratory geese (Lyons et al. 2024), which strongly influence vegetation (Uher‐Koch et al. 2019). These factors make the Y-K Delta an ideal system to investigate how warming, flooding, and herbivory, individually and in combination, shape plant functional trait expression, with findings likely relevant to other high-latitude coastal ecosystems facing similar environmental pressures.

In this study, we examined responses of four key plant functional traits related to size and leaf economics to warming, flooding, and herbivory in the wetlands of the Y-K Delta. Using a one-year mesocosm experiment in two distinct wetland plant communities, we applied a full-factorial manipulation of these global change drivers to assess immediate trait responses, reflecting trait plasticity, in three dominant species (two sedges and a deciduous dwarf-shrub). Because these drivers are expected to co-occur in the landscape, understanding whether their combined effects are additive or interactive is essential for predicting species responses. We focused on dominant species, whose traits disproportionately influence ecosystem functioning (Grime 1998) and selected two different functional groups to capture variation in life history strategies and resource acquisition. The presence of the same shrub species in both wetlands also allowed us to assess intraspecific trait plasticity across different biotic and abiotic conditions. We hypothesized that: (1) warming would generally increase plant size and favor acquisitive trait values, particularly in the deciduous shrub; (2) flooding would increase plant size and favor acquisitive trait values in graminoids but may reduce size and favor conservative traits in the shrub, indicative of stress responses; and (3) herbivory would reduce plant size while promoting acquisitive trait values, especially in graminoids.

## Methods

### Study area and wetland plant communities

We conducted this study in the Yukon Delta National Wildlife Refuge, a vast wetland system (~ 75,000 km^2^) formed by the Yukon and Kuskokwim rivers at their confluence with the Bering Sea (Appendix 1: Fig. S1). Our study sites were located 19 km inland from the Bering Sea and 45 river-km upstream from the mouth of the Kashunuk River. The climate is cold oceanic, with mean summer (June–August) and winter (January–March) temperatures of 12.5 °C and − 12.2 °C, respectively, and an average annual precipitation of 499 mm (30 year average for 1991–2020 at the Bethel weather station, ~ 200 km from the study site; Palecki et al. 2021). The plant growing season spans from late May to mid-August, while snow and ice typically cover the ground from October to early May.

We conducted the experiment in two distinct wetland plant communities, which we refer to as Lowland wetland (61° 26′ 10.93"N, 165° 26′ 20.98"W; 2.39 m elevation) and Upland wetland (61° 26′ 7.44"N, 165° 26′ 36.10"W; 2.56 m elevation). Both wetlands are characterized by fully saturated, oligohaline soils made of a relatively thick organic layer, and both experience occasional summer flooding. However, due to its slightly lower elevation, the Lowland wetland is subject to more frequent flooding, which contributes to differences in plant community composition and soil properties compared to the Upland wetland.

While both wetlands are dominated by *Carex* species and include dwarf-shrubs such as the relatively abundant *Salix fuscescens*, the subdominant *Betula nana*, and the forb *Potentilla palustris*, *C. rariflora* dominates the Lowland wetland, whereas *C. lyngbyei* dominates the Upland wetland. Soil properties also differ between the two wetlands: organic matter content (58% in Lowland vs. 40% in Upland), soluble salts (0.8 vs. 0.5 mmho cm^−1^), sodium (700 vs. 350 ppm), nitrate (0.18 vs. 0.10 ppm), phosphorus (10 vs. 14 ppm), and sulfate (130 vs. 70 ppm). In contrast, soil pH (~ 5.5) and C:N (~ 22) were similar in both wetlands (Ross 2024). These two communities are representative of the widespread wetland communities that compose the complex plant community mosaic of the Y-K Delta (Jorgenson 2000).

### Experimental design and treatments

We conducted the experiment within an 8 m × 16 m area in each of the two wetlands with homogeneous plant community composition and minimal topographic variation. At the end of the 2022 growing season, we established mesocosms by removing 60 cm × 41 cm × 18 cm (length × width × height) soil and vegetation monoliths and placing them into transparent plastic containers of the same dimensions. These containers were reinserted into the ground at the same location from which the monoliths had been extracted (Appendix 1: Fig. S2). Each wetland contained 60 mesocosms, for a total of 120 across both sites. Mesocosms were arranged in five rows of 12, with 1 m spacing in all directions. The setup was conducted the year before measurements to allow the monoliths time to adjust to the experimental conditions. Treatment implementation and data collection took place in summer 2023.

Within each row, treated separately to avoid spatial clustering of treatment combinations by chance, we randomly assigned mesocosms to a full-factorial combination of three treatments: warming (ambient temperature or warming), flooding (no flooding, low-intensity flooding, or high-intensity flooding), and herbivory (no herbivory or herbivory), resulting in 12 treatment combinations. To exclude natural herbivory, we fenced both wetland areas during summer 2023. No flying herbivores (geese) or evidence of geese were ever observed in the enclosed areas. In three replicates of each treatment combination per wetland (36 mesocosms per wetland), we recorded air (+ 10 cm) and soil (− 5 cm) temperature every 90 min from early June to mid-August using dataloggers (iButtons DS1921G/Z, Maxim Integrated, San Jose, California, USA) (Appendix 1: Figs. S3-S4).

#### Warming treatment

To increase temperature, we installed ITEX-style (Hollister et al. 2022), conical open-top chambers (OTCs; 85 cm base × 50 cm top × 35 cm height; Kalwall Corp., Manchester, New Hampshire, USA) from 09-Jun-2023 to 11-Aug-2023. During the summer, OTCs increased mean air temperature by ~ 1.2 °C and soil temperature by ~ 0.2 °C. The increase in air temperature aligns with projected summer warming in the Y-K Delta, beyond warming that has already occurred, for the period 2040–2049 (SNAP 2020). At our site, OTCs had negligible effects on soil moisture, air relative humidity, and free-air CO_2_ concentration (Petit Bon et al. 2024).

#### Flooding treatment

To simulate flooding, we added high-tide water during three flooding events in mid-June, early July, and late July. The water source for the Lowland wetland was a small slough located approximately 50 m from the mesocosms, while in the Upland wetland we used a larger slough located about 30 m away.

We applied two flooding intensities to simulate different levels of high-tide inundation. Low-intensity flooding represented two consecutive days of overbank high-tide flooding per month, while high-intensity flooding represented six consecutive days of overbank high-tide flooding per month. These durations were based on projected future high-tide levels from relative sea-level rise forecasts for western Alaska (Sweet et al. 2022) and on the established relationship between tidal dynamics in our study area and the nearest tidal monitoring station in Nome (~ 330 km away; Terenzi et al. 2014). In the Lowland wetland, low-intensity flooding corresponds to conditions projected for the late 2020s, and high-intensity flooding to the mid-2030s. In the Upland wetland, low-intensity flooding is expected by the mid-2040s, and high-intensity flooding by the late 2050s (see Petit Bon et al. 2024 for further details).

On each flooding day that we measured, we added water until it reached the top of each mesocosm, on average 8.3 ± 2.6 SD L across all days and flooding events. We did not drain mesocosms because flooding treatments were meant to wet soils not only during flooding events but thereafter as would occur during a natural flooding event. Between flooding events, transpiration and evaporation reduced water levels to such an extent that we added approximately the same amount of water the first day of each flooding event, with rainfall likely accounting for differences. During flooding events, we added about 2–3 L to each mesocosm per day, signifying containers were more saturated during flooding events than between each flooding event, but there was significant loss each day to transpiration and evaporation even during flooding events.

We collected floodwater samples at the beginning and end of each consecutive flooding day and flooding event and calculated seasonal averages for total dissolved solids (1.08 ± 0.36 g L^−1^ in Lowland and 0.70 ± 0.64 g L^−1^ in Upland) and pH (6.8 ± 0.4 and 7.5 ± 0.3). These values were consistent with measurements from high-tide water samples collected daily throughout the summer (total dissolved solids: 0.84 ± 0.75 g L^−1^; pH: 7.1 ± 0.3) using a multiparameter water meter (HI98194, Hanna Instruments, Woonsocket, Rhode Island, USA).

#### Herbivory treatment

To apply the herbivory treatment, we simulated the three main components of goose herbivory: (1) grazing, (2) fecal deposition, and (3) trampling. The treatment was based on the intensity of herbivore pressure exerted by *Branta nigricans* (Pacific black brant), a common grazer in coastal wetlands. As low-lying coastal habitats become increasingly inundated, geese are expected to shift inland, applying similar grazing pressure to currently less-impacted areas such as our study wetlands. Therefore, our herbivory treatment represents current levels of herbivory in more heavily grazed coastal areas.

To simulate grazing, we clipped vegetation by removing leaves, buds, and fruits from both herbaceous and woody plants, as well as some herbaceous stems, until reaching the biomass target. Because goose diets can include substantial non-graminoid tissues, such as leaf tissue of other plants and berries from shrubs (Sedinger and Raveling 1984; Cadieux et al. 2005), we included these parts in our clipping protocol. Prior to clipping, we quantified the percent cover of each plant functional group (graminoids, deciduous dwarf-shrubs, evergreen dwarf-shrubs, and forbs) in each mesocosm using the point-intercept method (Bråthen and Hagberg 2004) with a 7 × 7 crossed grid (49 points). To reflect that more abundant species are likely grazed more frequently, we clipped each functional group in proportion to its relative cover (cf. Choi et al. 2022), thereby applying uniform grazing pressure relative to availability. This also allowed for meaningful comparison of plant trait responses among functional groups. We removed 26.4 g m^−2^ dry weight of aboveground biomass per mesocosm, corresponding to ~ 10% of peak-season biomass (Petit Bon et al. 2024), consistent with mean seasonal offtake by geese (Person et al. 1998). Clipping occurred twice during the season: once in late June and again in mid-to-late July. Biomass removal was greater in the second event (17.6 g m^−2^ vs. 8.8 g m^−2^) to reflect increased post-hatch grazing pressure, when goslings grow rapidly and adults molt in preparation for fall migration.

To simulate fecal deposition, we added 27 goose feces m^−2^ in mid-June, following the average natural deposition rate for *B. nigricans* throughout the summer (Beard et al. 2023). We collected fresh feces from the study area and distributed them evenly within each mesocosm. Feces were applied once early in the season to allow for prolonged nutrient effects and to ensure presence during trampling.

To simulate trampling, we applied pressure equivalent to the average body mass of *B. nigricans* (~ 1.2 kg), which exerts approximately 15.5 g cm^−2^ per step. We applied this load twice: after fecal addition and again following the second grazing event.

### Focal species and functional trait sampling

We assessed aboveground plant trait responses to warming, flooding, and herbivory for the two dominant species in each wetland: *C. rariflora* and *S. fuscescens* in the Lowland wetland, and *C. lyngbyei* and *S. fuscescens* in the Upland wetland. This sampling design allowed us to compare (1) the magnitude of trait responses between species from different functional groups (sedges and deciduous dwarf-shrubs) within a given wetland, which are known to differ inherently in trait values (Chapin et al. 1996; Thomas et al. 2018), and (2) the response of a single species (*S. fuscescens*) growing under different biotic and abiotic conditions in the two wetlands.

Trait sampling was conducted between 31-Jul-2023 and 5-Aug-2023. From each mesocosm, we collected focal traits from two fully mature individuals of each focal species, selected at random, except for *C. rariflora* (Lowland wetland), for which we sampled three individuals. In total, we sampled 616 individuals. From each *Carex* individual, two leaves were collected; from each *S. fuscescens* individual, three leaves were collected. Leaves were pooled per individual for trait measurements. For *S. fuscescens*, leaves were selected from sun-exposed positions at or above the graminoid canopy, following standardized protocols (Pérez-Harguindeguy et al. 2013).

To isolate the direct effects of herbivory, we only sampled individuals that exhibited evidence of grazing in herbivory-treated mesocosms. For *Carex* spp., this meant leaves visibly clipped. For *S. fuscescens*, we sampled intact leaves from individuals that had other leaves removed during the herbivory treatment. This distinction reflects typical goose foraging behavior: graminoids are grazed at the leaf tip, leaving a portion intact, whereas shrubs with smaller, non-elongated leaves are more likely to experience full leaf removal.

We focused on four widely used aboveground plant traits, representing the two major axes of plant functional trait variation (Díaz et al. 2016). We measured two size-related traits, vegetative height (cm) and leaf area (mm^2^), and two resource economics traits, specific leaf area (SLA; mm^2^ g^−1^) and leaf dry matter content (LDMC; g g^−1^). While we acknowledge that leaf thickness, which can be more plastic than LDMC and vary independently, may provide complementary information on leaf structure, we focused on LDMC because it is related to thickness (Blumenthal et al. 2020) and the SLA-LDMC continuum is often used to capture the main axis of leaf economic strategies. Plant height was measured in the field on undisturbed individuals as the vertical distance from the ground to the highest vegetative tissue. After harvest, leaves were kept moist in zip-lock bags with dampened paper towels and refrigerated for a maximum of 48 h before processing. We first recorded fresh mass, then scanned leaves (Perfection V600 Photo Scanner, Epson America Inc., Los Alamitos, California, USA) and calculated leaf area using ImageJ (Schneider et al. 2012). Leaves were then oven-dried at 60 °C to constant mass and weighed. SLA was calculated by dividing leaf area by dry mass, and LDMC by dividing fresh mass by dry mass. Although LDMC is defined using water-saturated fresh mass (Pérez-Harguindeguy et al. 2013), leaf rehydration was not feasible under our field conditions at this remote sampling location. Yet, our approach ensures consistent comparisons within our experiment and aligns with procedures commonly used at remote tundra sites (e.g., Jónsdóttir et al. 2022; Kemppinen & Niittynen 2022).

### Statistical analysis

We conducted all analyses in R v. 4.4.0 (https://www.r-project.org/). For each species, we averaged trait values across individuals within each mesocosm prior to analysis. We used the ‘*vegan*’ package to conduct permutational multivariate analysis of variance (PERMANOVA; Oksanen et al. 2020), the ‘*prcomp*’ function in base R for principal component analysis (PCA), and the ‘*glmmTMB*’ package (Brooks et al. 2017) to fit generalized linear mixed-effects models (GLMMs). We used the ‘*car*’ package (Fox & Weisberg 2018) for ANOVA and the ‘*emmeans*’ package (Lenth 2021) to calculate marginal means and perform pairwise comparisons. Model fit was assessed using the ‘*DHARMa*’ package (Hartig 2024), and lognormal transformations were applied when necessary to improve residual normality and homoscedasticity. We used the ‘*ggplot2*’ package (Wickham 2016) and the ‘*factoextra*’ package (Kassambara 2020) for data visualization.

To explore inherent differences in trait values among species of different plant functional groups and potential intraspecific variation in *S. fuscescens* across wetlands, we first tested for interspecific trait differences, treating *S. fuscescens* in the two wetlands as distinct entities. To assess multivariate trait differences, we performed a PERMANOVA using species as a fixed-effect on the centered and scaled Euclidean distance matrix of the four traits (height, leaf area, SLA, and LDMC). Row of mesocosms was included as a random-effect. Results were visualized using PCA. Complementarily, we tested each trait individually using GLMMs with species as a fixed-effect and row as a random-effect. We assessed species differences using Type III Wald chi-square tests via ANOVA and performed pairwise comparisons using Tukey-adjusted contrasts.

To evaluate treatment effects on multivariate trait expression, we conducted a separate PERMANOVA for each species and wetland type, using the same approach described above. Fixed-effects included warming, flooding, herbivory, and all two- and three-way interactions; row was included as a random-effect. Because none of the interactions were significant (*P* > 0.05), we report results from models including only the main effects. Multivariate trait responses were visualized via PCA, and loadings and scores of individual treatment effects were displayed separately for interpretability.

To examine treatment effects on individual traits, we used a separate GLMM for each trait, species, and wetland. Fixed-effects included warming, flooding, herbivory, and their interactions; row was included as a random-effect. When highest-order interactions were not significant, we sequentially removed the least significant term until either all retained interactions were significant or only main effects remained. We assessed treatment differences using Type III Wald chi-square tests via ANOVA and performed pairwise comparisons. For flooding (a three-level factor), we conducted pairwise contrasts between pre-specified levels (low-intensity vs. no flooding and high-intensity vs. no flooding); as only these planned comparisons were tested, no correction for multiple comparisons was applied (Gelman and Hill 2007). We present z-values and p-values from the conditional model to show all flooding levels.

## Results

### Interspecific functional trait differences

**Fig. 1:**
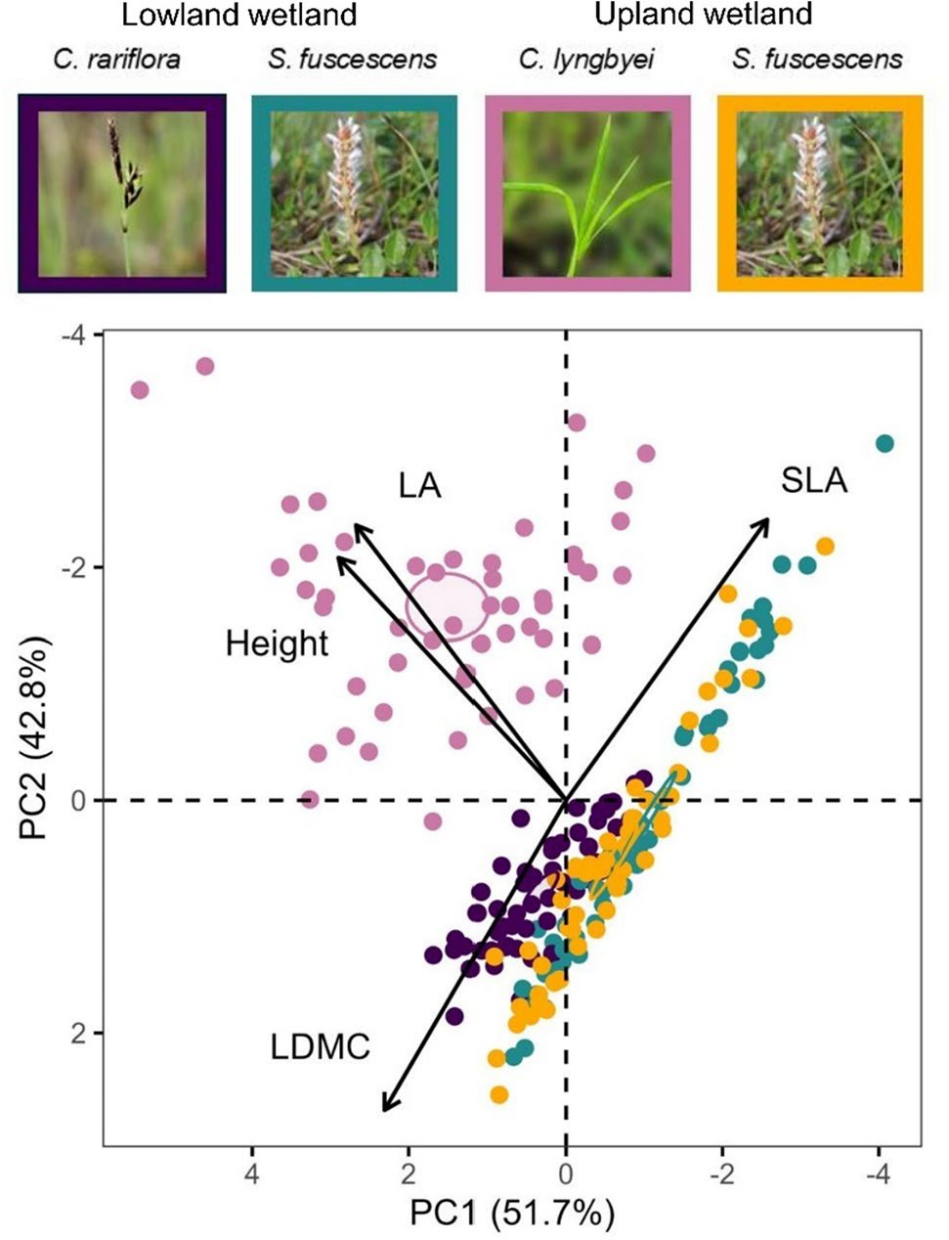
Plant species in the multivariate functional trait space. PCA biplot of plant traits: height, leaf area (LA), specific leaf area (SLA), and leaf dry matter content (LDMC), shown as black arrows. Each point represents the PCA scores of one species in a mesocosm and is colored by species identity. *S. fuscescens* is shown separately for the two wetlands. Ellipses indicate 95% confidence intervals around species centroids. Percent variance explained by the first two principal components is shown on the axes

Plant species differed significantly in their multivariate trait expression (PERMANOVA: F_3,216_ = 60.2, *P* < 0.0001; Fig. 1, Appendix 1: Table S1). The PCA indicated that the sedge *C. lyngbyei* was associated with the greatest vegetative height and leaf area, *C. rariflora* with the highest LDMC and lowest SLA, and the deciduous dwarf-shrub *S. fuscescens* with generally lower height and leaf area, and higher SLA and lower LDMC compared to the sedge *C. rariflora*.

Accordingly, species also differed significantly in individual trait values (Appendix 1: Table S2). The three species differed in height (*P* < 0.001) in the order *C. lyngbyei* > *C. rariflora* > *S. fuscescens*, with no height difference between *S. fuscescens* individuals in the two wetlands. All pairwise differences in leaf area were significant among species, including between *S. fuscescens* in the Lowland and Upland wetlands (*P* < 0.05), with *C. lyngbyei* having the largest leaf area, followed by *C. rariflora*, *S. fuscescens* in the Upland wetland, and *S. fuscescens* in the Lowland wetland. *C. rariflora* had lower SLA than all other species (*P* < 0.001), while *C. lyngbyei* and *S. fuscescens* (in both wetlands) did not differ in SLA. LDMC was higher in *C. rariflora* than in *C. lyngbyei* and *S. fuscescens* in the Lowland wetland (*P* < 0.001), but not different from *S. fuscescens* in the Upland wetland. *S. fuscescens* in the Upland wetland had greater LDMC than in the Lowland wetland, while *C. lyngbyei* did not differ from either.

### Treatment effects on plant functional traits

**Fig. 2:**
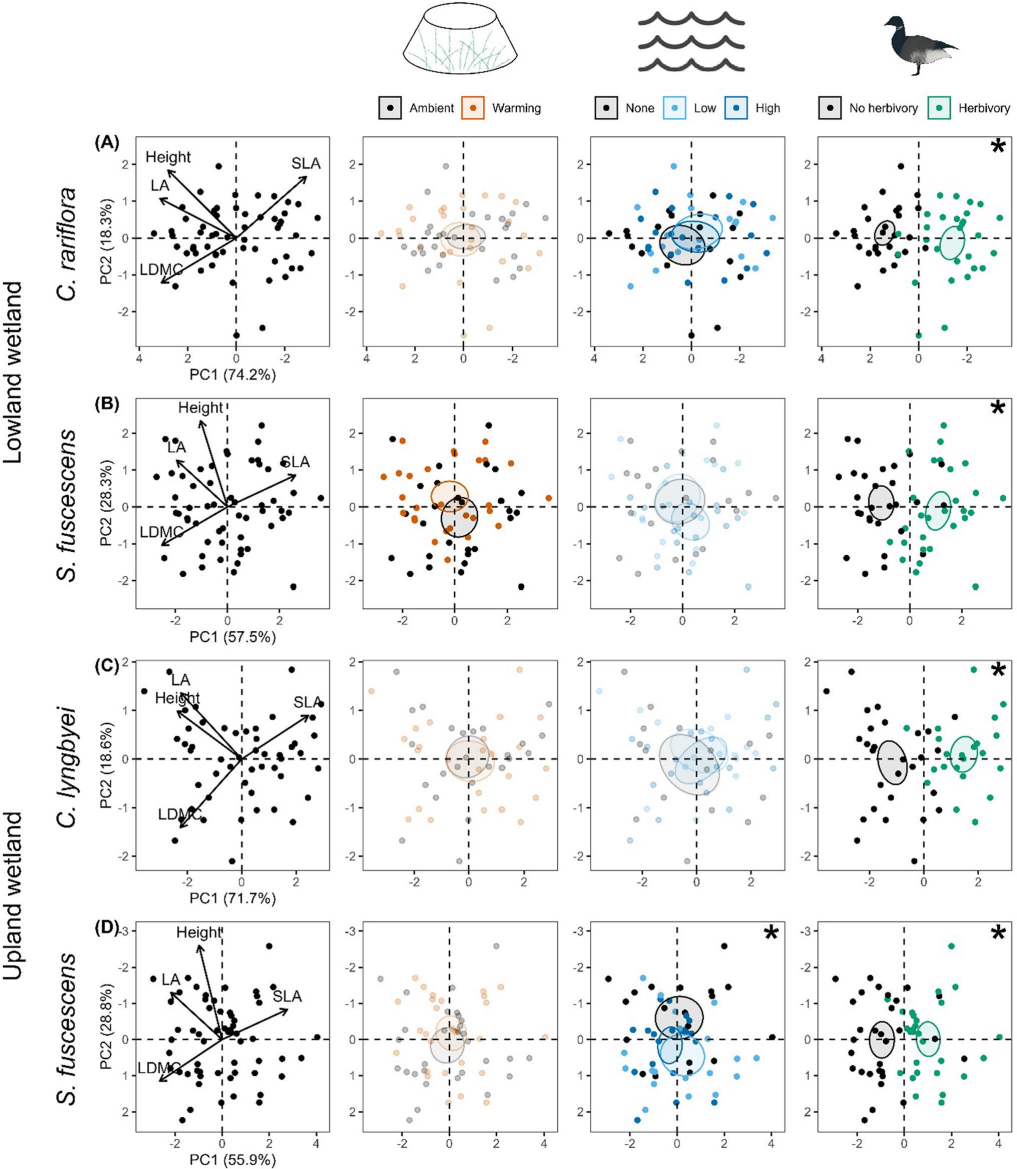
Effects of treatments on multivariate functional trait expression. PCA biplots and PERMANOVA results of plant traits under warming, flooding (low- and high-intensity), and herbivory. Panels show results for: (**A**) *C. rariflora*, (**B**) *S. fuscescens* in the Lowland wetland, (**C**) *C. lyngbyei*, and (**D**) *S. fuscescens* in the Upland wetland. While treatment-specific biplots are displayed separately for interpretability, PCA and PERMANOVA were conducted once per species. Ellipses indicate 95% confidence intervals around treatment centroids. The four focal traits are shown as black arrows in the left column, which depicts the general trait structure for each species. Biplots are bolded when treatment effects were marginally significant (*P* < 0.10) and marked with asterisks when significant (*P* < 0.05). Percent variance explained by the first two principal components is shown on the axes in the left column

**Fig. 3:**
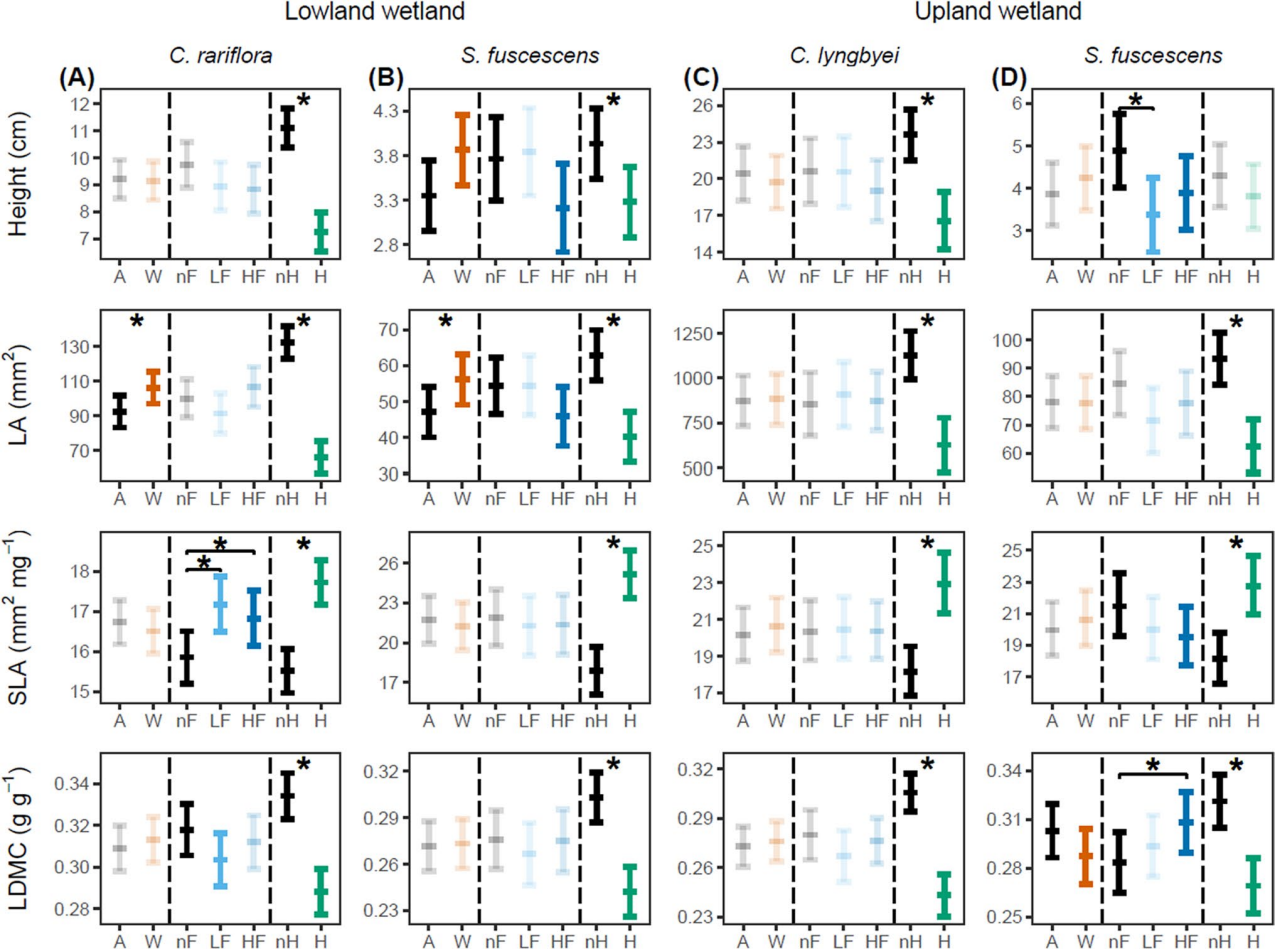
Main effects of treatments on individual functional traits. GLMM predictions (± 95% confidence intervals) of plant traits under warming, flooding, and herbivory. Panels show modeled means for each species: (**A**) *C. rariflora*, (**B**) *S. fuscescens* in the Lowland wetland, (**C**) *C. lyngbyei*, and (**D**) *S. fuscescens* in the Upland wetland. Treatment levels include ambient temperature (A), warming (W), no flooding (nF), low-intensity flooding (LF), high-intensity flooding (HF), no herbivory (nH), and herbivory (H). Means and confidence intervals were back-transformed to the response scale for visualization. Bolded values indicate marginally significant effects (*P* < 0.10); asterisks denote significance at *P* < 0.05. Note that *y*-axis scales differ across species. For *S. fuscescens* in the Upland wetland, the interaction between flooding and warming on vegetative height is not visualized here; see Fig. 4 for details

The multivariate trait space of *C. rariflora* was not affected by warming, but was affected by flooding (PERMANOVA: F_2,51_ = 2.5, *P* = 0.07) and strongly by herbivory (F_1,51_ = 56.6, *P* < 0.001; Fig. 2A, Appendix 1: Table S3). However, at the single-trait level (Fig. 3A, Appendix 1: Table S4), warming increased leaf area by 15% (*z* = 2.1, *P* < 0.05). Low- and high-intensity flooding increased SLA by 8% (*z* = 2.8, *P* < 0.01) and 6% (*z* = 2.1, *P* < 0.05), respectively. Low-intensity flooding also decreased LDMC by 6% (*z* = − 1.9, *P* = 0.06). Herbivory reduced height by 35% (*z* = − 7.7, *P* < 0.001), leaf area by 50% (*z* = − 10.1, *P* < 0.001), and LDMC by 12% (*z* = − 7.3, *P* < 0.001), while increasing SLA by 14% (*z* = 5.6, *P* < 0.001).

For *S. fuscescens* in the Lowland wetland, the multivariate trait space was affected by warming (PERMANOVA: F_1,51_ = 2.3, *P* = 0.09) and strongly affected by herbivory (F_1,51_ = 24.6, *P* < 0.001), but not by flooding (Fig. 2B, Appendix 1: Table S3). At the single-trait level (Fig. 3B, Appendix 1: Table S4), warming increased height by 15% (*z* = 1.9, *P* = 0.06) and leaf area by 19% (*z* = 2.2, *P* < 0.05). High-intensity flooding decreased height by 15% (*z* = − 1.7, *P* < 0.10) and leaf area by 16% (*z* = − 1.7, *P* = 0.09). Herbivory decreased height by 17% (*z* = − 2.4, *P* < 0.05), leaf area by 36% (*z* = − 5.5, *P* < 0.001), and LDMC by 20% (*z* = − 5.3, *P* < 0.001), and increased SLA by 40% (*z* = 5.8, *P* < 0.001).

The multivariate trait space of *C. lyngbyei* was affected only by herbivory (PERMANOVA: F_1,45_ = 36.1, *P* < 0.001), with no effects of warming or flooding (Fig. 2C, Appendix 1: Table S3). At the single-trait level (Fig. 3C, Appendix 1: Table S4), herbivory decreased height by 30% (*z* = − 4.6, *P* < 0.001), leaf area by 44% (*z* = − 5.0, *P* < 0.001), and LDMC by 23% (*z* = − 7.3, *P* < 0.001), and increased SLA by 27% (*z* = 6.7, *P* < 0.001). Warming and flooding had no effects on any individual trait.

**Fig. 4:**
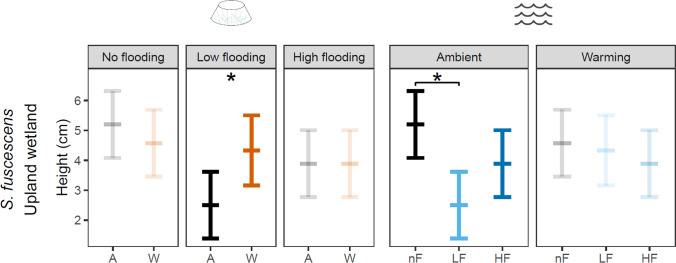
Two-way interaction between flooding and warming on vegetative height of *S. fuscescens* in the Upland wetland. GLMM predictions (± 95% confidence intervals) under combinations of flooding and temperature treatments. Treatment levels include ambient temperature (A), warming (W), no flooding (nF), low-intensity flooding (LF), and high-intensity flooding (HF). Bolded values indicate marginally significant effects (*P* < 0.10); asterisks denote significance at *P* < 0.05

For *S. fuscescens* in the Upland wetland, the multivariate trait space was affected by flooding (PERMANOVA: F_2,53_ = 2.6, *P* = 0.03) and herbivory (F_1,53_ = 19.7, *P* < 0.001), but not by warming (Fig. 2D, Appendix 1: Table S3). However, at the single-trait level (Fig. 3D, Appendix 1: Table S4), warming decreased LDMC by 16% (*z* = − 1.9, *P* = 0.06). High-intensity flooding decreased SLA by 9% (*z* = − 1.8, *P* = 0.07) and increased LDMC by 11% (*z* = 2.4, *P* < 0.05). Herbivory decreased leaf area by 33% (*z* = − 4.7, *P* < 0.001) and LDMC by 16% (*z* = − 6.2, *P* < 0.001), and increased SLA by 25% (*z* = 5.2, *P* < 0.001). There was a significant warming × flooding interaction for plant height: flooding reduced height under ambient conditions, while warming increased height, but only under low-intensity flooding (*z* = 2.4, *P* < 0.05; Fig. 4).

## Discussion

Using a mesocosm experiment in two high-latitude coastal wetlands of the Y-K Delta, differing in landscape position and associated biotic and abiotic characteristics, we assessed the trait plasticity of three dominant plant species in response to multiple global change drivers. A single summer of simulated warming, flooding, and herbivory was sufficient to induce rapid changes in key aboveground functional traits, indicating a pronounced short-term capacity for phenotypic adjustment. These drivers acted largely additively, with limited evidence for interactive effects. However, the overall responsiveness and magnitude of trait plasticity varied across drivers and species. Herbivory consistently exerted the strongest influence on both size-related and leaf economics traits across all species, while responses to flooding and warming were more variable and dependent on species and wetland communities. This combination of consistent and context-dependent responses underscores the complexity of trait adjustment to multiple environmental changes and provides an important step toward predicting how high-latitude coastal wetlands may respond functionally to a changing climate.

Our finding that warming increased vegetative height (+ 15%) and leaf area (+ 15–19%) of *C. rariflora* and *S. fuscescens* in the Lowland wetland supports our prediction that higher temperatures promote increases in size-related trait values, indicative of greater biomass. This aligns with warming experiments showing positive effects on plant size in high-latitude ecosystems (Hudson et al. 2011; Baruah et al. 2017; Wei et al. 2023) and globally (Dobson and Zarnetske 2025), as well as with observational studies across latitudinal, elevational, and micro-topographical temperature gradients (Betway et al. 2021; Kemppinen and Niittynen 2022; Simin et al. 2022). However, no warming effects were detected in *C. lyngbyei* or *S. fuscescens* in the Upland wetland. Betway et al. (2021) similarly observed that while most tundra species increased in size across a 7 °C latitudinal gradient, others remained relatively stable. Stability may reflect inherently low trait plasticity but could also indicate “diminishing returns” on investment in size, whereby further allocation to structural growth yields limited functional benefits and constrains responsiveness to warming (Niklas et al. 2007). Such constraints may also help explain the limited response of *C. lyngbyei* to flooding. Similarly, Baruah et al. (2017) found that the deciduous dwarf-shrub *S. herbacea* increased in height in dry and tussock tundra but not in wet meadows when exposed to higher temperatures, suggesting that wetland context may have constrained *S. fuscescens* responses in the Upland wetland. In our study, this could be partly explained by competition or shading from *C. lyngbyei*, which is five times taller and produces ten times the leaf area of *S. fuscescens*. Despite predictions that warming would promote more acquisitive trait values, especially in the shrub, leaf economics traits showed minimal responses, except for a slight decrease in LDMC in *S. fuscescens* in the Upland wetland. While this weak response contrasts our expectations, it fits within broader mixed evidence: although increased acquisitive traits are the most reported response to warming across the Arctic (Bjorkman et al. 2018; Jessen et al. 2020; Wei et al. 2023), some studies have found shifts toward more conservative trait values (Hudson et al. 2011). Our findings suggest that leaf economic traits may be less responsive to short-term warming than size-related traits and are strongly shaped by species- and context-dependent plasticity (Hudson et al. 2011; Kemppinen and Niittynen 2022).

In line with our hypothesis, high-intensity flooding reduced vegetative height and leaf area of *S. fuscescens* in the Lowland wetland by 15% and 16%, respectively, consistent with negative impacts of inundation on shrub growth (Person and Ruess 2003; Churchill et al. 2015) and with broader patterns of height decline along flooding gradients (Baastrup-Spohr et al. 2015; Fu et al. 2015; but see Purcell et al. 2019). In the Upland wetland, both flooding intensities decreased *S. fuscescens* height under ambient but not warmed conditions, suggesting that warming may buffer flooding-induced growth reductions in certain contexts. Also, in line with our hypothesis, high-intensity flooding altered resource economics traits, increasing LDMC (+ 11%) and decreasing SLA (− 9%) in *S. fuscescens* in the Upland wetland, indicating a shift toward more conservative trait values under stress. Conversely, *C. rariflora* in the Lowland wetland exhibited a more acquisitive response, with increased SLA (+ 6%) and decreased LDMC (− 6%), the latter occurring only under low-intensity flooding. These contrasting species-specific responses mirror findings from lower-latitude systems, where flooding selects conservative or acquisitive strategies depending on species and context (Violle et al. 2011; Purcell et al. 2019), with plasticity further contributing to the dominant trait expression (Jung et al. 2010). In our system, the conservative response of the slower-growing dwarf-shrub contrasted with the more acquisitive response of the faster-growing sedge, which is better adapted to inundation. These findings support the potential role of flooding in reinforcing graminoid dominance in these high-latitude wetlands (Petit Bon et al. 2024). Notably, flooding effects on resource-use traits were evident in *C. rariflora* but absent in *C. lyngbyei*, and in *S. fuscescens* only in the Upland wetland, highlighting both species-specific responses, even among congeners, and the fundamental role of environmental context in shaping trait plasticity under new flooding regimes.

Herbivory, simulated as goose grazing, feces addition, and trampling, triggered a coordinated trait response across all three species, consistently affecting all four functional traits and emerging as the only driver to elicit a response in the otherwise unresponsive sedge *C. lyngbyei*. In line with our hypothesis, herbivory reduced vegetative height (17–35%) and leaf area (33–50%), aligning with known negative effects of grazing on plant size (Díaz et al. 2001; Barbero-Palacios et al. 2024). In sedges, these reductions likely resulted from direct defoliation, which in our study overcame any new growth typically observed following herbivory (Cargill and Jefferies 1984; Beard et al. 2019). In the deciduous dwarf-shrub *S. fuscescens*, the decline in size-related traits may instead reflect an avoidance response (Díaz et al. 2007; Jessen et al. 2020), and trampling, which could contribute to reduced height and constrained leaf development, as shrubs are particularly sensitive to physical disturbance (Egelkraut et al. 2020; Tuomi et al. 2021). Grazing may have also kept *S. fuscescens* in earlier vegetative stages. The observed increases in SLA (14–40%) and decreases in LDMC (12–23%), suggest that plants in these wetlands generally resist herbivory by investing in rapid regrowth (Díaz et al. 2001; Niu et al. 2016), consistent with our hypothesis. This also indicates for economic traits that *S. fuscescens* did not adopt a conservative strategy for herbivore avoidance. Nutrient input from feces may have further enhanced SLA, in line with fertilization experiments (Iturrate-Garcia et al. 2020; Jessen et al. 2020). Because high SLA is associated with rapid growth and high photosynthetic rates (Wright et al. 2004; Reich 2014), our results suggest that dominant species in high-latitude coastal wetlands maintain trait values that sustain performance and competitive ability under herbivory.

Warming, flooding, and herbivory primarily affected focal plant functional traits through additive rather than interactive effects. However, species varied in their sensitivity to these drivers. *C. rariflora* was highly responsive: flooding and herbivory together shifted this sedge toward more acquisitive trait values, with both drivers increasing SLA and decreasing LDMC, resulting in one of the stronger changes in leaf economics among all species. In contrast, warming and herbivory had opposing effects on size-related traits in both *C. rariflora* and *S. fuscescens* in the Lowland wetland, indicating that higher temperatures may partially offset the strong reduction in plant size caused by herbivory. These observations highlight the need to consider the additive effects of multiple global change drivers, which may reinforce or offset each other, in shaping the magnitude and direction of immediate trait change. Thus, even in the absence of interactions, each driver contributed significantly to trait variation, underscoring the need for multifactorial approaches to better predict the functional consequences of climate change in Arctic coastal ecosystems.

In summary, our one-year study demonstrates that warming, flooding, and herbivory – three major drivers of environmental change in high-latitude coastal wetlands of the Y-K Delta and beyond – can elicit immediate trait responses in dominant plant species. Herbivory reduced plant size and promoted acquisitive trait values, even in the deciduous dwarf-shrub *S. fuscescens*, which is thought to be less herbivore-tolerant than the two sedges (*C. rariflora* and *C. lyngbyei*). This pattern for the deciduous shrub may reflect the tendency of high-latitude deciduous plants to rapidly maximize carbon gain (Johnson and Tieszen 1976); shifting to a more acquisitive strategy under herbivory could indicate their ability to further accelerate growth when stressed. Flooding emerged as a stronger control than warming, reducing the size of *S. fuscescens* and shifting economics trait values in line with species-specific flood tolerance: toward conservative values in the less flood-tolerant *S. fuscescens* and acquisitive values in the more flood-tolerant *C. rariflora*. However, *C. lyngbyei* did not respond as *C. rariflora* did, and economics trait shifts in *S. fuscescens* were detected only in the Upland, not the Lowland, wetland. These results underscore the importance of understanding both intra- and interspecific trait responses across varying biotic and abiotic contexts to better predict future shifts in plant functional composition. Phenotypic adjustments at these levels may mediate plant community responses and ecosystem functioning even without species turnover (Siefert et al. 2015; Henn et al. 2018; Westerband et al. 2021; Jónsdóttir et al. 2022). While we focused on commonly used aboveground traits central to plant performance and biogeochemical processes (Bjorkman et al. 2018), belowground trait responses particularly to flooding, remain largely unknown (Purcell et al. 2019; Freschet et al. 2021; Pan et al. 2022). Expanding our work to include belowground trait dimensions will be essential for improving predictions of plant community and ecosystem responses to multiple, co-occurring global change drivers in coastal high-latitude wetlands.

## Supplementary Information

Below is the link to the electronic supplementary material.

Supplementary file1 (DOCX 8361 KB)

## Data Availability

The data that support the findings of this study are openly available in the Arctic Data Center depository at 10.18739/A2M03Z02F.

## References

[CR1] Baastrup-Spohr L, Sand-Jensen K, Nicolajsen SV, Bruun HH (2015) From soaking wet to bone dry: predicting plant community composition along a steep hydrological gradient. J Veg Sci 26(4):619–630. 10.1111/jvs.12280

[CR2] Barbero-Palacios L, Barrio IC, García Criado M, Kater I, Petit Bon M, Kolari THM et al (2024) Herbivore diversity effects on Arctic tundra ecosystems: a systematic review. Environ Evid 13:6. 10.1186/s13750-024-00330-9 39294685 10.1186/s13750-024-00330-9 PMC11378771

[CR3] Barrio IC, Vuorinen KEM, Barbero-Palacios L, Defourneaux M, Petit Bon M, Greer EA et al (2025) Emerging priorities in terrestrial herbivory research in the Arctic. Arct Sci 11:1–26. 10.1139/as-2024-0080

[CR4] Baruah G, Molau U, Bai Y, Alatalo JM (2017) Community and species-specific responses of plant traits to 23 years of experimental warming across subarctic tundra plant communities. Sci Rep 7:2571. 10.1038/s41598-017-02595-2 28566722 10.1038/s41598-017-02595-2 PMC5451416

[CR5] Beard KH, Choi RT, Leffler AJ, Carlson LG, Kelsey KC, Schmutz JA et al (2019) Migratory goose arrival time plays a larger role in influencing forage quality than advancing springs in an Arctic coastal wetland. PLoS ONE 14(3):e0213037. 10.1371/journal.pone.0213037 30865725 10.1371/journal.pone.0213037 PMC6415786

[CR6] Beard KH, Kelsey KC, Choi RT, Welker JM, Leffler AJ (2023) Goose feces effects on subarctic soil nitrogen availability and greenhouse gas fluxes. Ecosystems 26:187–200. 10.1007/s10021-022-00752-x

[CR7] Beccari E, Carmona CP (2024) Aboveground and belowground sizes are aligned in the unified spectrum of plant form and function. Nat Commun 15:9199. 10.1038/s41467-024-53180-x 39448582 10.1038/s41467-024-53180-x PMC11502772

[CR8] Betway KR, Hollister RD, May JL, Oberbauer SF (2021) Species-specific trends and variability in plant functional traits across a latitudinal gradient in northern Alaska. J Veg Sci 32(3):e13040. 10.1111/jvs.13040

[CR9] Bjorkman AD, Myers-Smith IH, Elmendorf SC, Normand S, Rüger N, Beck PSA et al (2018) Plant functional trait change across a warming tundra biome. Nature 562:57–62. 10.1038/s41586-018-0563-7 30258229 10.1038/s41586-018-0563-7

[CR10] Blumenthal DM, Mueller KE, Kray JA, Ocheltree TW, Augustine DJ, Wilcox KR (2020) Traits link drought resistance with herbivore defence and plant economics in semi-arid grasslands: the central roles of phenology and leaf dry matter content. J Ecol 108(6):2336–2351. 10.1111/1365-2745.13454

[CR11] Bråthen KA, Hagberg O (2004) More efficient estimation of plant biomass. J Veg Sci 15(5):653–660. 10.1111/j.1654-1103.2004.tb02307.x

[CR12] Brooks ME, Kristensen K, van Benthem KJ, Magnusson A, Berg CW, Nielsen A et al (2017) GlmmTMB balances speed and flexibility among packages for zero-inflated generalized linear mixed modeling. R J 9(2):378–400. 10.32614/RJ-2017-066

[CR13] Cadieux M-C, Gauthier G, Hughes RJ (2005) Feeding ecology of Canada geese (*Branta canadensis interior*) in sub-Arctic inland tundra during brood-rearing. Auk 122(1):144–157. 10.1093/auk/122.1.144

[CR14] Cargill SM, Jefferies RL (1984) The effects of grazing by lesser snow geese on the vegetation of a sub-Arctic salt marsh. J Appl Ecol 21(2):669–686. 10.2307/2403437

[CR15] Chapin FS, Bret-Harte MS, Hobbie SE, Zhong H (1996) Plant functional types as predictors of transient responses of arctic vegetation to global change. J Veg Sci 7(3):347–358. 10.2307/3236278

[CR16] Choi RT, Petit Bon M, Leffler AJ, Kelsey KC, Welker JM, Beard KH (2022) Short-term effects of experimental goose grazing and warming differ in three low-Arctic coastal wetland plant communities. J Veg Sci 33(3):e13139. 10.1111/jvs.13139

[CR17] Churchill AC, Turetsky MR, McGuire AD, Hollingsworth TN (2015) Response of plant community structure and primary productivity to experimental drought and flooding in an Alaskan fen. Can J Forest Res 45(2):185–193. 10.1139/cjfr-2014-0100

[CR18] Díaz S, Noy-Meir I, Cabido M (2001) Can grazing response of herbaceous plants be predicted from simple vegetative traits? J Appl Ecol 38(3):497–508. 10.1046/j.1365-2664.2001.00635.x

[CR19] Díaz S, Lavorel S, McIntyre S, Falczuk V, Casanoves F, Milchunas DG et al (2007) Plant trait responses to grazing – a global synthesis. Glob Change Biol 13(2):313–341. 10.1111/j.1365-2486.2006.01288.x

[CR20] Díaz S, Kattge J, Cornelissen JHC, Wright IJ, Lavorel S, Dray S et al (2016) The global spectrum of plant form and function. Nature 529:167–171. 10.1038/nature16489 26700811 10.1038/nature16489

[CR21] Dobson KC, Zarnetske PL (2025) A global meta-analysis of passive experimental warming effects on plant traits and community properties. Glob Change Biol 31(6):e70306. 10.1111/gcb.70306 10.1111/gcb.70306 PMC12183621 40546057

[CR22] Egelkraut D, Barthelemy H, Olofsson J (2020) Reindeer trampling promotes vegetation changes in tundra heathlands: results from a simulation experiment. J Veg Sci 31(3):476–486. 10.1111/jvs.12871

[CR23] Elmendorf SC, Henry GHR, Hollister RD, Björk RG, Bjorkman AD, Callaghan TV et al (2012) Global assessment of experimental climate warming on tundra vegetation: heterogeneity over space and time. Ecol Lett 15(2):164–175. 10.1111/j.1461-0248.2011.01716.x 22136670 10.1111/j.1461-0248.2011.01716.x

[CR24] Fox AD, Madsen J (2017) Threatened species to super-abundance: the unexpected international implications of successful goose conservation. Ambio 46:179–187. 10.1007/s13280-016-0878-2 28215012 10.1007/s13280-016-0878-2 PMC5316321

[CR25] Fox J, Weisberg S (2018) An R companion to applied regression, 3rd ed. Sage Publications

[CR26] Freschet GT, Roumet C, Comas LH, Weemstra M, Bengough AG, Rewald B et al (2021) Root traits as drivers of plant and ecosystem functioning: current understanding, pitfalls and future research needs. New Phytol 232(3):1123–1158. 10.1111/nph.17072 33159479 10.1111/nph.17072

[CR27] Fu H, Zhong J, Yuan G, Guo C, Ding H, Feng Q et al (2015) A functional-trait approach reveals community diversity and assembly processes responses to flood disturbance in a subtropical wetland. Ecol Res 30:57–66. 10.1007/s11284-014-1207-5

[CR84] Grime JP (1998) Benefits of plant diversity to ecosystems: immediate filter and founder effects. J Ecol 86(6):902–910. 10.1046/j.1365-2745.1998.00306.x

[CR28] Gelman A, Hill J (2007) Data analysis using regression and multilevel/hierarchical models. Cambridge University Press

[CR29] Hamlington BD, Bellas-Manley A, Willis JK, Fournier S, Vinogradova N, Nerem RS et al (2024) The rate of global sea level rise doubled during the past three decades. Commun Earth Environ 5:601. 10.1038/s43247-024-01761-5

[CR30] Hartig F (2024) “DHARMa: residual diagnostics for hierarchical (multi-level/mixed) regression models (Version 0.4.7) [R]”. Accessed 30 Jun 2025 from https://CRAN.R-project.org/package=DHARMa

[CR31] Henn JJ, Buzzard V, Enquist BJ, Halbritter AH, Klanderud K, Maitner BS et al (2018) Intraspecific trait variation and phenotypic plasticity mediate alpine plant species response to climate change. Front Plant Sci 9:1548. 10.3389/fpls.2018.01548 30483276 10.3389/fpls.2018.01548 PMC6243391

[CR32] Hollister RD, Elphinstone C, Henry GHR, Bjorkman AD, Klanderud K, Björk RG et al (2022) A review of open top chamber (OTC) performance across the ITEX Network. Arctic Sci 9(2):331–344. 10.1139/as-2022-0030

[CR33] Hudson JMG, Henry GHR, Cornwell WK (2011) Taller and larger: shifts in Arctic tundra leaf traits after 16 years of experimental warming. Glob Chang Biol 17(2):1013–1021. 10.1111/j.1365-2486.2010.02294.x

[CR34] IPCC (2023) Climate change 2023: the physical science basis. Contribution of Working Groups I, II and III to the Sixth Assessment Report of the Intergovernmental Panel on Climate Change. Cambridge University Press

[CR35] Irrgang AM, Bendixen M, Farquharson LM, Baranskaya AV, Erikson LH, Gibbs AE et al (2022) Drivers, dynamics and impacts of changing Arctic coasts. Nat Rev Earth Environ 3:39–54. 10.1038/s43017-021-00232-1

[CR36] Iturrate-Garcia M, Heijmans MMPD, Cornelissen JHC, Schweingruber FH, Niklaus PA, Schaepman-Strub G (2020) Plant trait response of tundra shrubs to permafrost thaw and nutrient addition. Biogeosciences 17(20):4981–4998. 10.5194/bg-17-4981-2020

[CR37] Jessen MT, Kaarlejärvi E, Olofsson J, Eskelinen A (2020) Mammalian herbivory shapes intraspecific trait responses to warmer climate and nutrient enrichment. Glob Change Biol 26(12):6742–6752. 10.1111/gcb.15378 10.1111/gcb.15378 33020977

[CR38] Johnson DA, Tieszen LL (1976) Aboveground biomass allocation, leaf growth, and photosynthesis patterns in tundra plant forms in arctic Alaska. Oecologia 24:159–173. 10.1007/BF00572757 28309333 10.1007/BF00572757

[CR39] Jónsdóttir IS, Halbritter AH, Christiansen CT, Althuizen IHJ, Haugum SV, Henn JJ et al (2022) Intraspecific trait variability is a key feature underlying high Arctic plant community resistance to climate warming. Ecol Monogr 93(1):e1555. 10.1002/ecm.1555

[CR40] Jorgenson MT (2000) Hierarchical organization of ecosystems at multiple spatial scales on the Yukon-Kuskokwim Delta, Alaska, USA. Arct Antarct Alp Res 32(3):221–239. 10.1080/15230430.2000.12003360

[CR41] Jorgenson MT, Ely C (2001) Topography and flooding of coastal ecosystems on the Yukon-Kuskokwim Delta, Alaska: implications for sea-level rise. J Coast Res 17(1):124–136

[CR42] Jung V, Violle C, Mondy C, Hoffmann L, Muller S (2010) Intraspecific variability and trait-based community assembly. J Ecol 98(5):1134–1140. 10.1111/j.1365-2745.2010.01687.x

[CR43] Kassambara A (2020) "Factoextra: extract and visualize the results of multivariate data analyses (Version 1.0.7) [R]". Accessed 30 Jun 2025 from https://CRAN.R-project.org/package=factoextra

[CR44] Kemppinen J, Niittynen P (2022) Microclimate relationships of intraspecific trait variation in sub‐Arctic plants. Oikos 2022(12):e09507. 10.1111/oik.09507

[CR45] Kirezci E, Young IR, Ranasinghe R, Muis S, Nicholls RJ, Lincke D et al (2020) Projections of global-scale extreme sea levels and resulting episodic coastal flooding over the 21st Century. Sci Rep 10:11629. 10.1038/s41598-020-67736-6 32732976 10.1038/s41598-020-67736-6 PMC7393110

[CR46] Koltz AM, Gough L, McLaren JR (2022) Herbivores in Arctic ecosystems: effects of climate change and implications for carbon and nutrient cycling. Ann N Y Acad Sci 1516(1):28–47. 10.1111/nyas.14863 35881516 10.1111/nyas.14863 PMC9796801

[CR47] Lameris TK, Hoekendijk J, Aarts G, Aarts A, Allen AM, Bienfait L et al (2021) Migratory vertebrates shift migration timing and distributions in a warming Arctic. Anim Migr 8(1):110–131. 10.1515/ami-2020-0112

[CR48] Lenth RV (2021) "emmeans: estimated marginal means, aka least-squares means (Version 1.5.4) [R]". Accessed 30 Jun 2025 from https://CRAN.R-project.org/package=emmeans

[CR49] Lyons JE, Brown SC, Saalfeld ST, Johnson JA, Andres BA, Sowl KM et al (2024) Alaska’s climate sensitive Yukon–Kuskokwim Delta supports seven million Arctic-breeding shorebirds, including the majority of six North American populations. Ornithol Appl 126(2):duad066. 10.1093/ornithapp/duad066

[CR50] Myers-Smith IH, Thomas HJD, Bjorkman AD (2019) Plant traits inform predictions of tundra responses to global change. New Phytol 221(4):1742–1748. 10.1111/nph.15592 30444539 10.1111/nph.15592

[CR51] Nicholls RJ, Lincke D, Hinkel J, Brown S, Vafeidis AT, Meyssignac B et al (2021) A global analysis of subsidence, relative sea-level change and coastal flood exposure. Nat Clim Chang 11:338–342. 10.1038/s41558-021-00993-z

[CR52] Nicotra AB, Atkin OK, Bonser SP, Davidson AM, Finnegan EJ, Mathesius U et al (2010) Plant phenotypic plasticity in a changing climate. Trends Plant Sci 15(12):684–692. 10.1016/j.tplants.2010.09.008 20970368 10.1016/j.tplants.2010.09.008

[CR53] Niklas KJ, Cobb ED, Niinemets Ü, Reich PB, Sellin A, Shipley B et al (2007) “Diminishing returns” in the scaling of functional leaf traits across and within species groups. Proc Natl Acad Sci USA 104(21):8891–8896. 10.1073/pnas.0701135104 17502616 10.1073/pnas.0701135104 PMC1885598

[CR54] Niu K, He J-S, Lechowicz MJ (2016) Grazing-induced shifts in community functional composition and soil nutrient availability in Tibetan alpine meadows. J Appl Ecol 53(5):1554–1564. 10.1111/1365-2664.12727

[CR55] Oksanen J, Blanchet FG, Friendly M, Kindt R, Legendre P, McGlinn D et al. (2020) "vegan: community ecology package (Version 2.5-7) [R]". Accessed 30 Jun 2025 from https://CRAN.R-project.org/package=vegan

[CR56] Palecki M., Durre I., Applequist S., Arguez A, Lawrimore J (2021) US climate normals 2020: US hourly climate normals (1991–2020). NOAA National Centers for Environmental Information. Accessed 30 Jun 2025 from https://gov.noaa.ncdc.C01622

[CR57] Pan Y, Cieraad E, Armstrong J, Armstrong W, Clarkson BR, Pedersen O et al (2022) Leading trait dimensions in flood-tolerant plants. Ann Bot 130(3):383–392. 10.1093/aob/mcac031 35259242 10.1093/aob/mcac031 PMC9486907

[CR58] Parker CL, Mooney PA, Webster MA, Boisvert LN (2022) The influence of recent and future climate change on spring Arctic cyclones. Nat Commun 13:6514. 10.1038/s41467-022-34126-7 36351898 10.1038/s41467-022-34126-7 PMC9646868

[CR59] Pérez-Harguindeguy N, Díaz S, Garnier E, Lavorel S, Poorter H, Jaureguiberry P et al (2013) New handbook for standardised measurement of plant functional traits worldwide. Aust J Bot 61:167–234. 10.1071/BT12225

[CR60] Person BT, Ruess RW (2003) Stability of a subarctic saltmarsh: plant community resistance to tidal inundation. Ecoscience 10(3):351–360. 10.1080/11956860.2003.11682784

[CR61] Person BT, Babcock CA, Ruess RW (1998) Forage variation in brood-rearing areas used by Pacific black brant geese on the Yukon-Kuskokwim Delta, Alaska. J Ecol 86(2):243–259

[CR62] Petit Bon M, Leffler AJ, Kelsey KC, Williams TJ, Beard KH (2024) Projected near-future flooding and warming increase graminoid biomass in a high-latitude coastal wetland. J Ecol 112(12):2715–2730. 10.1111/1365-2745.14418

[CR63] Purcell AS, Lee WG, Tanentzap AJ, Laughlin DC (2019) Fine root traits are correlated with flooding duration while aboveground traits are related to grazing in an ephemeral wetland. Wetlands 39:291–302. 10.1007/s13157-018-1084-8

[CR64] Rantanen M, Karpechko AY, Lipponen A, Nordling K, Hyvärinen O, Ruosteenoja K et al (2022) The Arctic has warmed nearly four times faster than the globe since 1979. Commun Earth Environ 3:168. 10.1038/s43247-022-00498-3

[CR65] Reich PB (2014) The world-wide ‘fast–slow’ plant economics spectrum: a traits manifesto. J Ecol 102(2):275–301. 10.1111/1365-2745.12211

[CR85] Ross JM (2024) The effects of temperature, flooding, and goose feces addition on greenhouse gas emissions and ammonification in four high-latitude soils from western Alaska. Master thesis, Department of Natural Resource Management, South Dakota State University, Brookings, South Dakota, USA

[CR66] Schneider CA, Rasband WS, Eliceiri KW (2012) NIH image to ImageJ: 25 years of image analysis. Nat Methods 9:671–675. 10.1038/nmeth.2089 22930834 10.1038/nmeth.2089 PMC5554542

[CR67] Sedinger JS, Raveling DG (1984) Dietary selectivity in relation to availability and quality of food for goslings of Cackling geese. Auk 101(2):295–306. 10.1093/auk/101.2.295

[CR68] Siefert A, Violle C, Chalmandrier L, Albert CH, Taudiere A, Fajardo A et al (2015) A global meta-analysis of the relative extent of intraspecific trait variation in plant communities. Ecol Lett 18(12):1406–1419. 10.1111/ele.12508 26415616 10.1111/ele.12508

[CR69] Simin T, Davie-Martin CL, Petersen J, Høye TT, Rinnan R (2022) Impacts of elevation on plant traits and volatile organic compound emissions in deciduous tundra shrubs. Sci Total Environ 837:155783. 10.1016/j.scitotenv.2022.155783 35537508 10.1016/j.scitotenv.2022.155783

[CR70] SNAP (2020) Scenarios network for Alaska and Arctic planning. University of Alaska, Fairbanks, CMIP5 model, RCP6.0 scenario comparison of 2010–19 to 2090–99

[CR71] Suding KN, Lavorel S, Chapin FS, Cornelissen JHC, Díaz S, Garnier E et al (2008) Scaling environmental change through the community-level: a trait-based response-and-effect framework for plants. Glob Change Biol 14(5):1125–1140. 10.1111/j.1365-2486.2008.01557.x

[CR72] Sweet WV, Hamlington BD, Kopp RE, Weaver CP, Barnard PL, Bekaert D et al. (2022) Global and regional sea level rise scenarios for the United States: Updated mean projections and extreme water level probabilities along US coastlines. National Oceanic and Atmospheric Administration

[CR73] Terenzi J, Jorgenson MT, Ely CR (2014) Storm-surge flooding on the Yukon-Kuskokwim delta, Alaska. Arctic 67(3):360–374

[CR74] Thomas HJD, Myers-Smith IH, Bjorkman AD, Elmendorf SC, Blok D, Cornelissen JHC et al (2018) Traditional plant functional groups explain variation in economic but not size-related traits across the tundra biome. Glob Ecol Biogeogr 28(2):78–95. 10.1111/geb.12783 31007605 10.1111/geb.12783 PMC6472633

[CR75] Tuomi M, Väisänen M, Ylänne H, Brearley FQ, Barrio IC, Bråthen KA et al (2021) Stomping in silence: conceptualizing trampling effects on soils in polar tundra. Funct Ecol 35(2):306–317. 10.1111/1365-2435.13719

[CR76] Uher-Koch BD, Schmutz JA, Wilson HM, Anthony RM, Day TL, Fondell TF et al (2019) Ecosystem-scale loss of grazing habitat impacted by abundance of dominant herbivores. Ecosphere 10(6):e02767. 10.1002/ecs2.2767

[CR77] Vermaire JC, Pisaric MF, Thienpont JR, Courtney Mustaphi CJ, Kokelj SV, Smol JP (2013) Arctic climate warming and sea ice declines lead to increased storm surge activity. Geophys Res Lett 40(7):1386–1390. 10.1002/grl.50191

[CR78] Violle C, Navas M-L, Vile D, Kazakou E, Fortunel C, Hummel I et al (2007) Let the concept of trait be functional! Oikos 116(5):882–892. 10.1111/j.0030-1299.2007.15559.x

[CR79] Violle C, Bonis A, Plantegenest M, Cudennec C, Damgaard C, Marion B et al (2011) Plant functional traits capture species richness variations along a flooding gradient. Oikos 120(3):389–398. 10.1111/j.1600-0706.2010.18525.x

[CR80] Wei B, Zhang D, Wang G, Liu Y, Li Q, Zheng Z et al (2023) Experimental warming altered plant functional traits and their coordination in a permafrost ecosystem. New Phytol 240(5):1802–1816. 10.1111/nph.19115 37434301 10.1111/nph.19115

[CR81] Westerband AC, Funk JL, Barton KE (2021) Intraspecific trait variation in plants: a renewed focus on its role in ecological processes. Ann Bot 4(1):397–410. 10.1093/aob/mcab011 10.1093/aob/mcab011 PMC7988520 33507251

[CR82] Wickham H (2016) Ggplot2: elegant graphics for data analysis. SpringerLink

[CR83] Wright IJ, Reich PB, Westoby M, Ackerly DD, Baruch Z, Bongers F et al (2004) The worldwide leaf economics spectrum. Nature 428:821–827. 10.1038/nature02403 15103368 10.1038/nature02403

