# Supplementary material for: Rapid plant functional trait responses to warming, flooding, and herbivory in high-latitude coastal wetlands
Source: Oecologia. 2026 Mar 8;208(3):40. doi: 10.1007/s00442-026-05876-8 (PMC12968112; doi:10.1007/s00442-026-05876-8)
Supplement: Supplementary file 1 — Supplementary file1 (DOCX 8361 KB) [file 442_2026_5876_MOESM1_ESM.docx]

**Supplementary Information: Appendix 1**

**Rapid plant functional trait responses to warming, flooding, and herbivory in high-latitude coastal wetlands,**

***Oecologia***

Cristina Chirvasa^1^, Matteo Petit Bon^1,5*^, Kelvyn K. Bladen^2^, Katharine C. Kelsey^3^, A. Joshua Leffler^4^, Tyler J. Williams^1^, and Karen H. Beard^1*^

^1^ Utah State Univ, Department of Wildland Resources and Ecology Center, Logan, UT USA

^2^ Utah State Univ, Department of Mathematics and Statistics, Logan, UT USA

^3^ Univ Colorado Denver, Department of Geography & Environmental Science, Denver, CO USA

^4^ South Dakota State Univ, Department of Natural Resource Management, Brookings, SD USA

^5^ Current address: North Carolina State Univ, Department of Applied Ecology, Raleigh, NC USA

* Matteo Petit Bon and Karen H. Beard should be considered joint senior authors

Corresponding Author: Cristina Chirvasa (cristina221b@yahoo.com)

**Supplementary tables**

**Table S1**.

PERMANOVA for all species across all traits and ANOVA for species by trait. † < 0.10; * < 0.05; ** < 0.01; *** < 0.001.

| **Predictor** | **Df** | | **SS** | | **R^2^** | | | **F** | | | **P-value** | |
| --- | --- | --- | --- | --- | --- | --- | --- | --- | --- | --- | --- | --- |
| Species | 3 | | 398.76 | | 0.455 | | | 60.159 | | | **0.0001***** | |
| Residual | 216 | | 477.24 | | 0.545 | | |  | | |  | |
| Total | 219 | | 876.00 | | 1 | | |  | | |  | |
|  |  | |  | | | |  | | |  |  |  |
| **Trait** | | **Treatment** | | **Df** | | **Chisqu** | | | **P-value** | | |  |
| Height | | (Intercept) | | 1 | | 690.91 | | | **<0.0001***** | | |  |
|  | | Species | | 3 | | 862.79 | | | **<0.0001***** | | |  |
| Leaf Area | | (Intercept) | | 1 | | 167.03 | | | **<0.0001***** | | |  |
|  | | Species | | 3 | | 2988.57 | | | **<0.0001***** | | |  |
| SLA | | (Intercept) | | 1 | | 1367.46 | | | **<0.0001***** | | |  |
|  | | Species | | 3 | | 33.16 | | | **<0.0001***** | | |  |
| LDMC | | (Intercept) | | 1 | | 2883.06 | | | **<0.0001***** | | |  |
|  | | Species | | 3 | | 27.26 | | | **<0.0001***** | | |  |

**Table S2**. Parameter estimates for species modelled means contrasts for each trait. Model estimates, standard error (SE), z-values, and p-values are obtained from generalized linear mixed models. Significant differences between treatments indicated in bold and as † < 0.10; * < 0.05; ** < 0.01; *** < 0.001.

| **Species contrast** | **Estimate** | **SE** | **Z-value** | **P-value** |
| --- | --- | --- | --- | --- |
| Height | | | | |
| *C. rariflora –* Lowland *S. fuscescens* | 5.53 | 0.40 | 13.78 | **< 0.0001***** |
| *C. rariflora – C. lyngbyei* | -8.31 | 0.59 | -14.04 | **< 0.0001***** |
| *C. rariflora –* Upland *S. fuscescens* | 5.28 | 0.43 | 12.39 | **< 0.0001***** |
| Lowland *S. fuscescens – C. lyngbyei* | -13.84 | 0.53 | -26.21 | **< 0.0001***** |
| Lowland *S. fuscescens –* Upland *S. fuscescens* | -0.25 | 0.34 | -0.73 | 0.88 |
| *C. lyngbyei –* Upland *S. fuscescens* | 13.59 | 0.54 | 25.17 | **< 0.0001***** |
| Leaf Area | | | | |
| *C. rariflora –* Lowland *S. fuscescens* | 45.5 | 8.3 | 5.51 | **< 0.0001***** |
| *C. rariflora – C. lyngbyei* | -579.7 | 13.1 | -44.14 | **< 0.0001***** |
| *C. rariflora –* Upland *S. fuscescens* | 22.2 | 8.4 | 2.63 | **0.04*** |
| Lowland *S. fuscescens – C. lyngbyei* | -625.3 | 11.6 | -53.98 | **< 0.0001***** |
| Lowland *S. fuscescens –* Upland *S. fuscescens* | -23.4 | 5.7 | -4.11 | **0.0002***** |
| *C. lyngbyei –* Upland *S. fuscescens* | 601.9 | 11.7 | 51.61 | **< 0.0001***** |
| Specific Leaf Area (SLA) | | | | |
| *C. rariflora –* Lowland *S. fuscescens* | -4.12 | 0.84 | -4.93 | **< 0.0001***** |
| *C. rariflora – C. lyngbyei* | -3.01 | 0.71 | -4.24 | **0.0001***** |
| *C. rariflora –* Upland *S. fuscescens* | -2.94 | 0.71 | -4.13 | **0.0002***** |
| Lowland *S. fuscescens – C. lyngbyei* | 1.11 | 0.86 | 1.30 | 0.57 |
| Lowland *S. fuscescens –* Upland *S. fuscescens* | 1.18 | 0.85 | 1.38 | 0.51 |
| *C. lyngbyei –* Upland *S. fuscescens* | 0.07 | 0.76 | 0.10 | 1.0 |
| Leaf Dry Matter Content (LDMC) | | | | |
| *C. rariflora –* Lowland *S. fuscescens* | 0.04 | 0.01 | 4.71 | **< 0.0001***** |
| *C. rariflora – C. lyngbyei* | 0.03 | 0.01 | 3.90 | **0.0007***** |
| *C. rariflora –* Upland *S. fuscescens* | 0.01 | 0.01 | 1.83 | 0.26 |
| Lowland *S. fuscescens – C. lyngbyei* | -0.01 | 0.01 | -0.67 | 0.91 |
| Lowland *S. fuscescens –* Upland *S. fuscescens* | -0.02 | 0.01 | -2.92 | **0.02*** |
| *C. lyngbyei –* Upland *S. fuscescens* | -0.02 | 0.01 | -2.15 | 0.14 |

**Table S3**. The effects of warming, low-intensity flooding, high-intensity flooding, and herbivory on each species per community. The effect of each treatment was tested by a permutational multivariate analysis of variance (PERMANOVA) performed across all traits. Abbreviations: SS = sum of squares, DF = degrees of freedom (numerator, denominator). Significant differences between treatments are indicated in bold and as † < 0.10; * < 0.05; ** < 0.01; *** < 0.001.

| **Species** | **Community** | **Predictor** | **SS** | **R^2^** | **F_(dfN, dfD)_** | **P-value** |
| --- | --- | --- | --- | --- | --- | --- |
| *C. rariflora* | Low Wet | Warming | 1.31 | 0.01 | 0.67 _(1,51)_ | 0.50 |
|  |  | Flooding | 9.63 | 0.04 | 2.48 _(2,51)_ | **0.07†** |
|  |  | Herbivory | 109.97 | 0.50 | 56.60 _(1,51)_ | **0.0001***** |
| *S. fuscescens* | Low Wet | Warming | 6.45 | 0.03 | 2.34 _(1,51)_ | **0.09†** |
|  |  | Flooding | 5.39 | 0.02 | 0.98 _(2,51)_ | 0.43 |
|  |  | Herbivory | 67.78 | 0.31 | 24.63 _(1,51)_ | **0.0001***** |
| *C. lyngbyei* | High Wet | Warming | 0.36 | 0.002 | 0.15 _(1,45)_ | 0.90 |
|  |  | Flooding | 2.36 | 0.01 | 0.49 _(2,45)_ | 0.74 |
|  |  | Herbivory | 85.99 | 0.44 | 36.07 _(1,45)_ | **0.0001***** |
| *S. fuscescens* | High Wet | Warming | 2.70 | 0.01 | 0.93 _(1,53)_ | 0.37 |
|  |  | Flooding | 15.02 | 0.07 | 2.60 _(2,53)_ | **0.03*** |
|  |  | Herbivory | 56.87 | 0.25 | 19.65 _(1,53)_ | **0.0001***** |

**Table S4**. Parameter estimates for treatment effect models (Table 2). Treatments are warming (W), low-intensity flooding (LF), high-intensity flooding (HF), and herbivory (H) and “:” signify interactions. Model estimates, standard error (SE), Z-values, and P-values are obtained from models in Table 1. Flooding treatments are compared to No Flooding. Significant differences between treatments are indicated in bold and as † < 0.10; * < 0.05; ** < 0.01; *** < 0.001.

| **Species** | **Community** | **Treatment** | **Estimate** | **SE** | **Z-value** | **P-value** |
| --- | --- | --- | --- | --- | --- | --- |
| Height | | | | | | |
| *C. rariflora* | Low Wet | W | -0.06 | 0.50 | -0.12 | 0.90 |
|  |  | LF | -0.80 | 0.61 | -1.30 | 0.19 |
|  |  | HF | -0.91 | 0.61 | -1.49 | 0.14 |
|  |  | H | -3.87 | 0.50 | -7.67 | **1.77E-14***** |
| *S. fuscescens* | Low Wet | W | 0.51 | 0.28 | 1.86 | **0.06†** |
|  |  | LF | 0.08 | 0.34 | 0.24 | 0.81 |
|  |  | HF | -0.55 | 0.34 | -1.65 | **0.0997†** |
|  |  | H | -0.65 | 0.28 | -2.36 | **0.02*** |
| *C. lyngbyei* | High Wet | W | -0.72 | 1.51 | -0.47 | 0.64 |
|  |  | LF | -0.07 | 1.90 | -0.04 | 0.97 |
|  |  | HF | -1.60 | 1.80 | -0.89 | 0.37 |
|  |  | H | -7.06 | 1.53 | -4.63 | **3.75E-6***** |
| *S. fuscescens* | High Wet | W | 0.37 | 0.43 | 0.86 | 0.39 |
|  |  | LF | -1.51 | 0.53 | -2.83 | **0.005**** |
|  |  | HF | -1.00 | 0.53 | -1.89 | **0.06†** |
|  |  | H | -0.48 | 0.43 | -1.12 | 0.26 |
| *S. fuscescens* | High Wet | W | -0.63 | 0.71 | -0.88 | 0.38 |
| Two-way |  | LF | -2.70 | 0.71 | -3.77 | **0.0002***** |
|  |  | HF | -1.31 | 0.71 | -1.83 | **0.07†** |
|  |  | H | -0.46 | 0.42 | -1.10 | 0.27 |
|  |  | W:LF | 2.45 | 1.03 | 2.39 | **0.02*** |
|  |  | W:HF | 0.63 | 1.01 | 0.62 | 0.54 |
| Leaf Area | | | | | | |
| *C. rariflora* | Low Wet | W | 13.80 | 6.51 | 2.12 | **0.03*** |
|  |  | LF | -8.67 | 7.92 | -1.10 | 0.27 |
|  |  | HF | 6.62 | 7.92 | 0.84 | 0.40 |
|  |  | H | -66.04 | 6.53 | -10.11 | **<2.00E-16***** |
| *S. fuscescens* | Low Wet | W | 9.04 | 4.10 | 2.20 | **0.03*** |
|  |  | LF | 0.09 | 5.00 | 0.02 | 0.99 |
|  |  | HF | -8.47 | 5.00 | -1.70 | **0.09†** |
|  |  | H | -22.70 | 4.12 | -5.51 | **3.51E-8***** |
| *C. lyngbyei* | High Wet | W | 9.63 | 98.92 | 0.10 | 0.92 |
|  |  | LF | 52.60 | 125.93 | 0.42 | 0.68 |
|  |  | HF | 17.97 | 118.97 | 0.15 | 0.88 |
|  |  | H | -500.45 | 99.93 | -5.01 | **5.50E-7***** |
| *S. fuscescens* | High Wet | W | -0.28 | 6.56 | -0.04 | 0.97 |
|  |  | LF | -13.02 | 8.07 | -1.61 | 0.11 |
|  |  | HF | -6.95 | 7.96 | -0.87 | 0.38 |
|  |  | H | -30.88 | 6.56 | -4.71 | **2.50E-6***** |
| Specific Leaf Area (SLA) | | | | | | |
| *C. rariflora* | Low Wet | W | -0.23 | 0.39 | -0.58 | 0.56 |
|  |  | LF | 1.32 | 0.47 | 2.79 | **0.005**** |
|  |  | HF | 0.97 | 0.47 | 2.05 | **0.04*** |
|  |  | H | 2.21 | 0.39 | 5.64 | **1.66E-8***** |
| *S. fuscescens* | Low Wet | W | -0.50 | 1.26 | -0.40 | 0.69 |
|  |  | LF | -0.60 | 1.54 | -0.39 | 0.69 |
|  |  | HF | -0.52 | 1.54 | -0.34 | 0.73 |
|  |  | H | 7.29 | 1.27 | 5.75 | **8.77E-9***** |
| *C. lyngbyei* | High Wet | W | 0.02 | 0.03 | 0.74 | 0.46 |
|  |  | LF | 0.01 | 0.04 | 0.14 | 0.89 |
|  |  | HF | 0.001 | 0.04 | 0.01 | 0.99 |
|  |  | H | 0.23 | 0.03 | 6.73 | **1.67E-11***** |
| *S. fuscescens* | High Wet | W | 0.03 | 0.04 | 0.75 | 0.45 |
|  |  | LF | -0.07 | 0.05 | -1.33 | 0.18 |
|  |  | HF | -0.10 | 0.05 | -1.84 | **0.07†** |
|  |  | H | 0.23 | 0.04 | 5.24 | **1.62E-7***** |
| Leaf Dry Matter Content (LDMC) | | | | | | |
| *C. rariflora* | Low Wet | W | 0.004 | 0.01 | 0.63 | 0.53 |
|  |  | LF | -0.01 | 0.01 | -1.88 | **0.06†** |
|  |  | HF | -0.01 | 0.01 | -0.77 | 0.44 |
|  |  | H | -0.05 | 0.01 | -7.34 | **2.18E-13***** |
| *S. fuscescens* | Low Wet | W | 0.001 | 0.01 | 0.13 | 0.90 |
|  |  | LF | -0.01 | 0.01 | -0.68 | 0.50 |
|  |  | HF | -0.001 | 0.01 | -0.06 | 0.95 |
|  |  | H | -0.06 | 0.01 | -5.33 | **9.72E-8***** |
| *C. lyngbyei* | High Wet | W | 0.003 | 0.01 | 0.37 | 0.71 |
|  |  | LF | -0.01 | 0.01 | -1.20 | 0.23 |
|  |  | HF | -0.004 | 0.01 | -0.37 | 0.71 |
|  |  | H | -0.06 | 0.01 | -7.32 | **2.57E-13***** |
| *S. fuscescens* | High Wet | W | -0.02 | 0.01 | -1.86 | **0.06†** |
|  |  | LF | 0.01 | 0.01 | 0.97 | 0.33 |
|  |  | HF | 0.02 | 0.01 | 2.41 | **0.02*** |
|  |  | H | -0.05 | 0.01 | -6.15 | **7.9E-10***** |

**Supplementary figures**


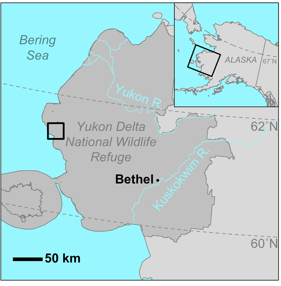

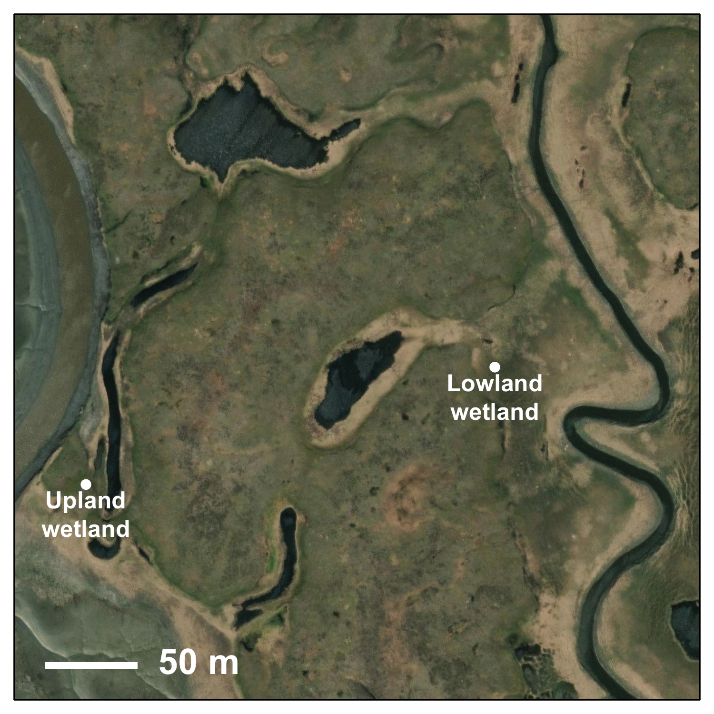


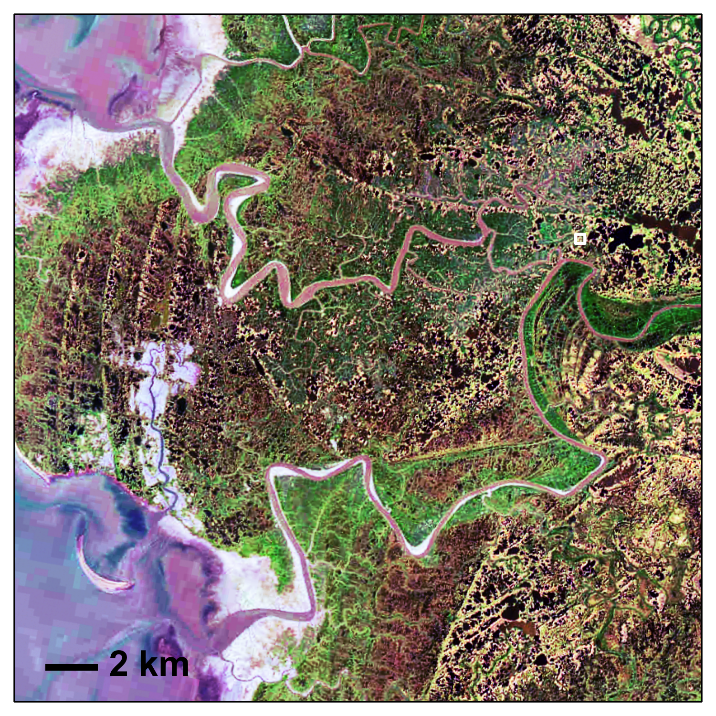

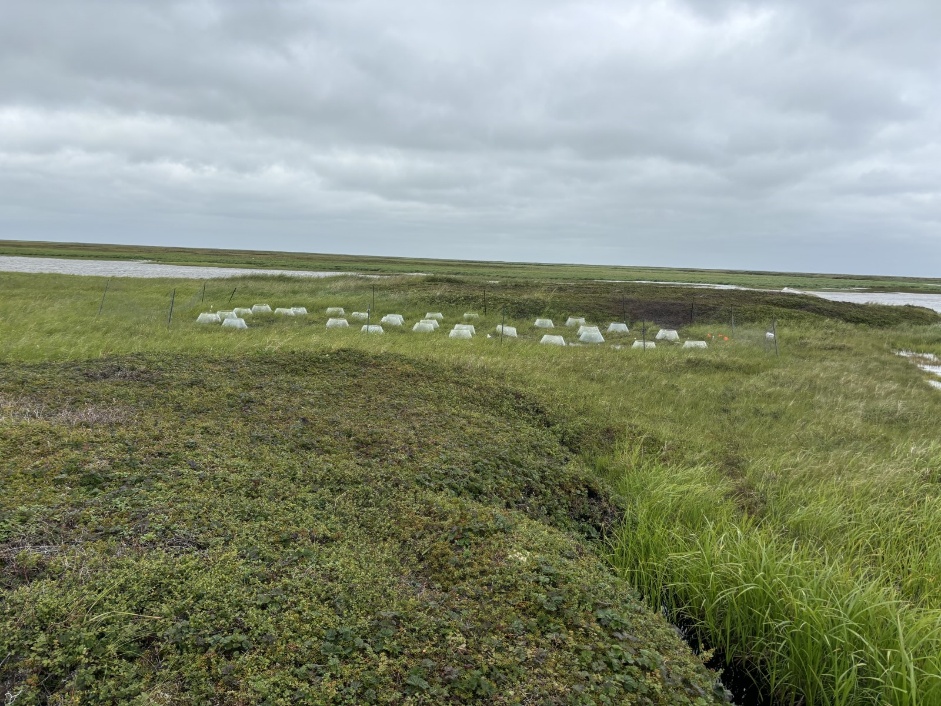


**(A)**

**(B)**

**(D)**

**(C)**

**Fig. S1.** Study site in the (A), Yukon-Kuskokwim Delta of Western Alaska, (B) region indicated with an arrow to a white square showing where samples were collected near the Keoklevik (north) and Kashunuk (south) rivers as seen in (C) the location of the two wetland communities, Lowland and Upland (61.436, -165.444; 61.435, -165.443), and (D) a photo of the Upland experiment. Maps and satellite images were obtained using the ‘basemaps’ package in R (Schwalb-Willmann 2024) from the ESRI (www.esri.com) ‘World Imagery’ collection dataset.


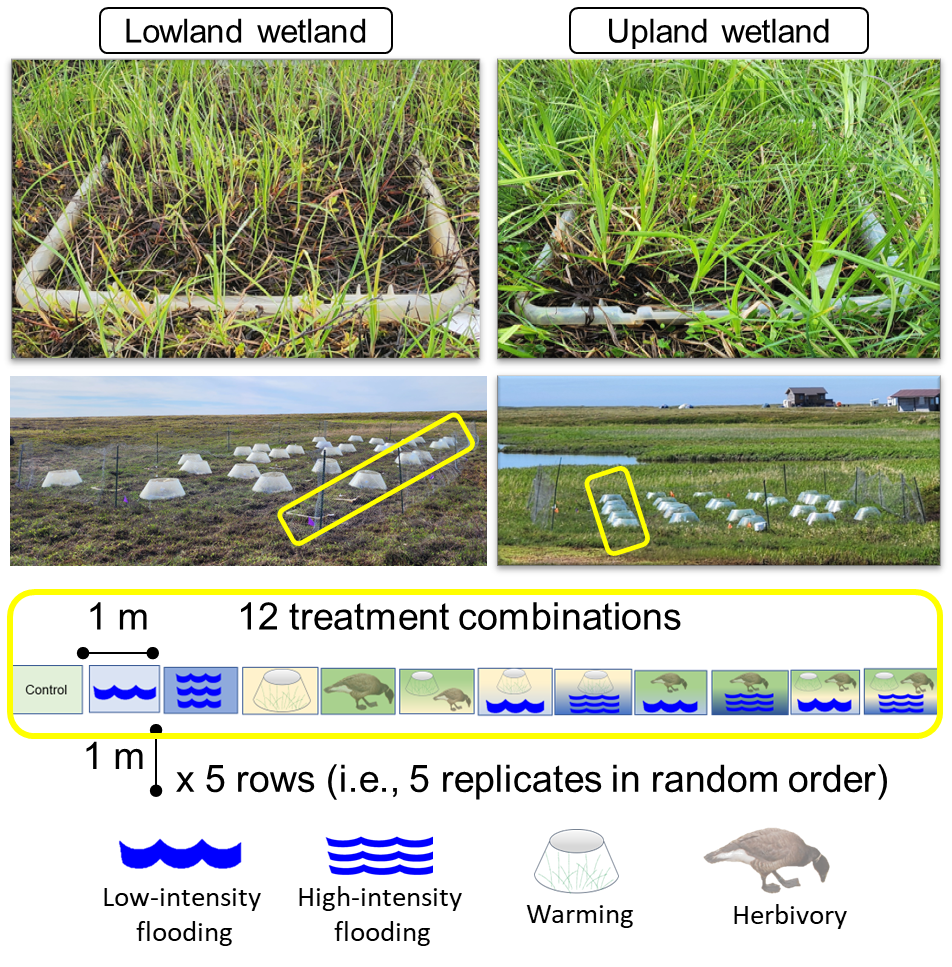


**(E)**

**(C)**

**(D)**

**(B)**

**(A)**

**Fig. S2.** Photos of the design (A) a close up of a Lowland wetland mesocosm, (B) an Upland wetland mesocosm, (C) early season Lowland wetland full design, (D) later in the season Upland wetland full design, and (E) illustrations of all 12 treatments used in the mesocosms, which included all combinations of flooding, warming, and herbivory. Treatments illustrations are described at the bottom. Not illustrated in the treatment schematic, but there was 1 m between each mesocosm in each direction. Mesocosm treatments were randomized across the 5 replicates but, when necessary, stratified to avoid having, by chance, spatially grouped treatments.


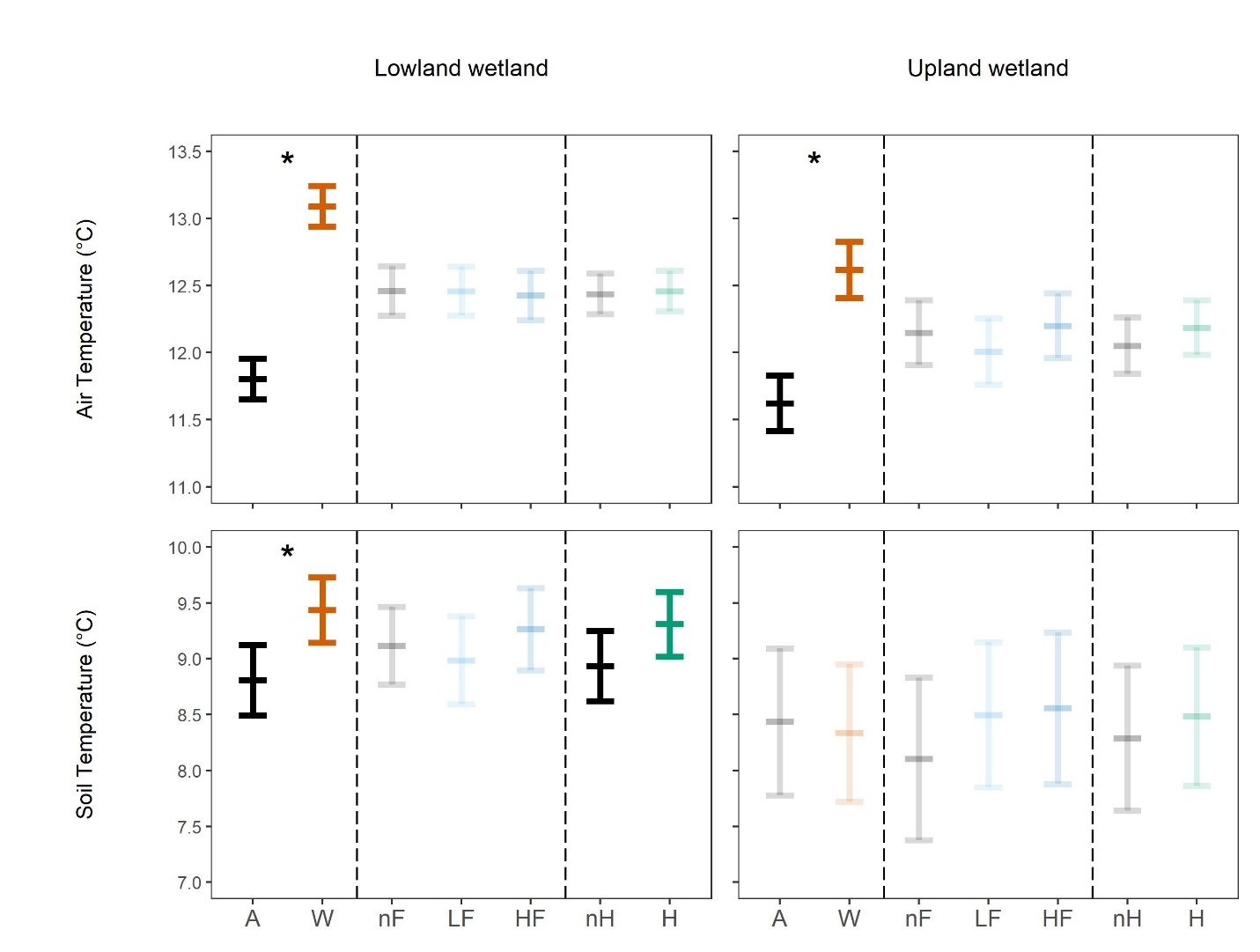


**(B)**

**(A)**

**Fig. S3.** Main effects of treatments on mesocosm-level temperatures. GLMM estimates (±95% confidence intervals) of air temperatures (upper panels) and soil temperatures (lower panels), separately for the two wetlands. Treatment levels include ambient temperature (A), warming (W), no flooding (nF), low-intensity flooding (LF), high-intensity flooding (HF), no herbivory (nH), and herbivory (H). Bolded values indicate marginally significant effects (*P* < 0.10); asterisks denote significance at *P* < 0.05. For soil temperatures in the Upland wetland, the detected interaction between flooding and warming is visualized in Fig. S4.


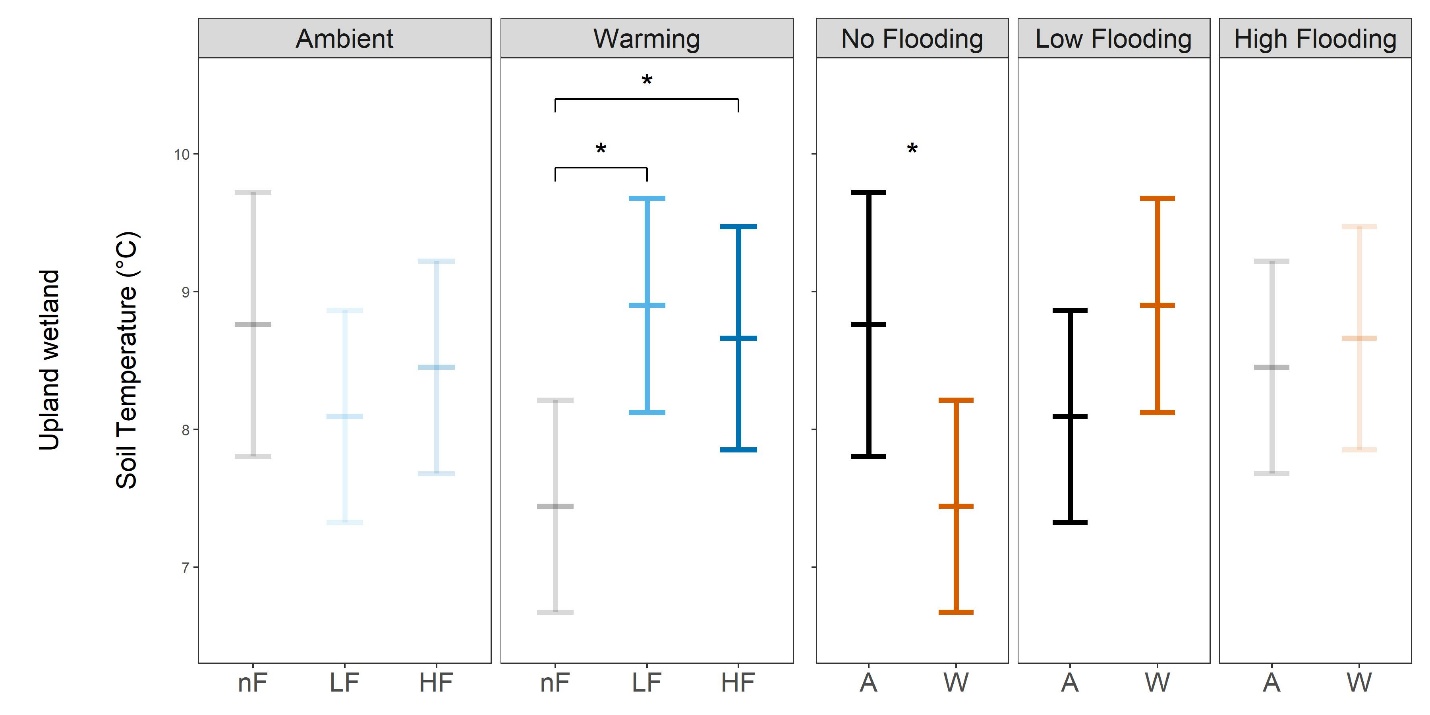


**Fig. S4**. Two-way interaction between flooding and warming on mesocosm-level soil temperatures in the Upland wetland. GLMM estimates (±95% confidence intervals) under combinations of flooding and temperature treatments. Treatment levels include ambient temperature (A), warming (W), no flooding (nF), low-intensity flooding (LF), and high-intensity flooding (HF). Bolded values indicate marginally significant effects (*P* < 0.10); asterisks denote significance at *P* < 0.05.
